# Supplementary material for: Two functionally distinct heme/iron transport systems are virulence determinants of the fish pathogen Flavobacterium psychrophilum
Source: Virulence. 2022 Jul 26;13(1):1221–41. doi: 10.1080/21505594.2022.2101197 (PMC9331221; doi:10.1080/21505594.2022.2101197)
Supplement: Supplemental Material [file KVIR_A_2101197_SM8529.docx]

**SUPPORTING INFORMATION**

[Supplementary Methods 2](#_Toc101776632)

[M1. Construction of the Δ*bfpR*, Δ*hfpR*, Δ*hfpY* and Δ*hfpR*Δ*bfpR* strains 2](#_Toc101776633)

[M2. Construction of pGi39 plasmid allowing chromosomal gene integration in *F. psychrophilum* and derivative reporter gene plasmids. 2](#_Toc101776634)

[M3. Construction of pGi39 derivative plasmids allowing complementation of *hfp* and *bfpR* deletion mutants. 2](#_Toc101776635)

[Supplementary Figures 3](#_Toc101776636)

[F1. Expression profiles of TonB-dependent receptors in *F. psychrophilum* OSU THCO2-90. 3](#_Toc101776637)

[F2. Schematic representation of the *F. psychrophilum* chromosomal gene expression platform and set of plasmids used for reporter genes expression and mutant complementation. 4](#_Toc101776638)

[F3. Validation of the chromosomal expression platform using reporter genes in strain OSU THCO2-90. 5](#_Toc101776639)

[F4. Transcriptional structure of *hfp* and *bfpR* loci analyzed by circular 3’-5′ RACE experiments. 6](#_Toc101776640)

[F5. Growth of *F. psychrophilum* OSU THCO2-90 at high concentration of hemoglobin. 8](#_Toc101776641)

[F6. RT-qPCR measurement of gene expression in complemented deletion mutants compared to wild-type. 8](#_Toc101776642)

[F7. Distinct *hfpR* and *hfpY* mRNA levels across several biological conditions assessed by microarrays. 9](#_Toc101776643)

[F8. THC0290_1813 encoding a predicted inner membrane protein of unknown function is not required for growth on hemoglobin as an iron source. 10](#_Toc101776644)

[F9. MSA of HfpY-like protein of *F. psychrophilum* with 5 characterized HmuY homologs. 11](#_Toc101776645)

[F10. Kaplan-Meier survival curves of rainbow trout after infection with wild-type, mutant and complemented strains. 12](#_Toc101776646)

[F11. MSA of HfpR and BfpR with other hemoglogin/heme TBDRs 13](#_Toc101776647)

[Supplementary Tables 16](#_Toc101776648)

[T1. Strains and plasmids. 16](#_Toc101776649)

[T2. List of oligonucleotides. 17](#_Toc101776650)

[T3. Genomic organization and predicted protein functions of the iron-induced gene cluster encoding the Hfp system in strain OSU THCO2-90. 19](#_Toc101776651)

[Supplementary references 20](#_Toc101776652)

Supplementary Methods

M1. Construction of the Δ*bfpR*, Δ*hfpR*, Δ*hfpY* and Δ*hfpR*Δ*bfpR* strains

Three suicide vectors pGi27, pGi29 and pGi38 allowing *hfpR*, *hfpY* and *bfpR* deletion respectively, were constructed using pYT313 plasmid [1] by three-fragments Gibson assembly using oligonucleotides listed in **Table** **T2**. For pGi27, three DNA fragments were amplified by PCR as follows: the plasmid fragment using pYT313 DNA as matrix and primers TRO186/TRO187, the 2,044-bp fragment upstream and 2,129-bp fragment downstream of *hfpR* with OSU THCO2-90 chromosomal DNA and primers TRO336/TRO337 and TRO338/TRO339, respectively. pGi29 and pGi38 were constructed similarly except that the 2-kb upstream and downstream fragments were amplified using primers TRO358/TRO359 and TRO360/TRO361 for *hfpY,* and TRO408/TRO409 and TRO410/TRO411 for *bfpR*.

Each resulting plasmid was introduced by conjugation in strain OSU THCO2-90 and colonies carrying chromosomal integration of the plasmid were selected using erythromycin resistance. The second recombination event (plasmid excision) was obtained by plating a stationary phase culture inoculated with a single colony in TYES broth without antibiotics on TYES supplemented with 5% sucrose. The chromosomal structure of erythromycin-sensitive clones was determined by PCR in order to identify the deletion mutant. The chromosomal structure of clones selected after double crossing over was analyzed by PCR using primers TRO341/TRO342 and TRO341/TRO325 for Δ*hfpR*, TRO379/TRO380 and TRO381/TRO382 for Δ*hfpY* and TRO417/TRO418 and TRO414/TRO415 for Δ*bfpR*. The double mutant Δ*hfpR*Δ*bfpR* was constructed by introduction of pGi38 plasmid by conjugation in Δ*hfpR*.

M2. Construction of pGi39 plasmid allowing chromosomal gene integration in *F. psychrophilum* and derivative reporter gene plasmids.

Several suicide vectors allowing chromosomal recombination and gene insertion at the neutral intergenic region were constructed using pYT313 plasmid [1] by Gibson method using oligonucleotides listed in **Table T2** (**Figure F2**).

pGi37 was designed to carry the 2,021-bp fragment upstream and the 1,979-bp fragment downstream of the insertion site (between positions 1121879 and 1124880 of OSU THCO2-90 genome) and a K7 composed of the 160-bp promoter region of *rpsL* gene (P*_rpsL_*), the *F. psychrophilum* codon adapted mCherry gene (mChFp; [2]) and an intrinsic terminator. Four DNA fragments were amplified by PCR as follows: the plasmid fragment using pYT313 DNA as matrix and primers TRO390/TRO419, the upstream and downstream fragments using OSU THCO2-90 chromosomal DNA and primers TRO420/TRO393 and TRO396/TRO397 respectively, and the P*_rpsL_*-mChFp K7 with pGi19 DNA and primers TRO394/TRO395. pGi19 is a pCP23-derivative plasmid carrying the mChFp gene under the control of the P*_rpsL_* promoter. pGi19 was constructed by assembly of the pCP23 fragment amplified using the pCP23-P*_rpsL_* plasmid and primers TRO245/TRO246 and the mChFp gene amplified using pUC-mChFp as matrix [2] and primers TRO247/248. pCP23-P*_rpsL_* was constructed by assembly of a pCP23 fragment amplified using primers TRO125/TRO126 and the P*_rpsL_* fragment amplified using primers TRO108/TRO111.

pGi39 was designed to carry the upstream and downstream fragments of the insertion site flanking a K7 composed of P*_rpsL_*, a multiple cloning site and an intrinsic terminator. pGi39 was constructed by Gibson assembly of the plasmid fragment amplified using pGi37 with primers TRO443/TRO444 and ssDNA primers TRO421/TRO422 (300 ng each) carrying the MCS sequence.

pGi42 was designed to allow chromosomal integration of the elastase gene FP0506 under the control of P*_rpsL_* at the insertion site. pGi42 was constructed by Gibson assembly of the plasmid fragment amplified using pGi37 DNA as matrix with primers TRO434/TRO435 and the FP0506 fragment amplified using JIP02/86 chromosomal DNA with primers TRO436/TRO437.

The chromosomal structure of clones selected after double crossing over was analyzed by PCR using primers TRO406/TRO407 and TRO402/TRO403.

M3. Construction of pGi39 derivative plasmids allowing complementation of *hfp* and *bfpR* deletion mutants.

Ectopic complementation of deletion mutants was performed by constructing a set of pGi39-derivative plasmids that allow gene insertion under the control of native promoter into the chromosome (**Figure F2**).

pGi44 that allows ectopic integration of the *hfpR* gene with the 240-bp upstream region was constructed as follows: the pGi39 fragment was amplified using pGi37 DNA as matrix without the P*_rpsL_*-mCh sequence with primers TRO443/TRO445 and the P*_hfpR_*-*hfpR* fragment using OSU THCO2-90 chromosomal DNA and primers TRO446/TRO447. Clones resulting from Gibson assembly were easily selected from false-positive clones (carrying pGi37 matrix) based on the absence of mCherry fluorescence signal.

pGi45 and pGi46 were designed to allow ectopic integration of the *bfpR* gene with the 306-bp upstream region (P*_bfpR_1_*_-_*bfpR*) and the two genes P*_bfpR_1_*-*bfpR*-P*_hfpR_*-*hfpR* in tandem, respectively. They were constructed by assembly of the pGi39 fragment amplified using pGi37 DNA as matrix and primers TRO443/TRO445 and, for pGi45, the P*_bfpR_1_*-*bfpR* fragment amplified using primers TRO448/TRO449, or for pGi46, the P*_bfpR_1_*-*bfpR* and P*_hfpR_*-*hfpR* fragments amplified using primers TRO448/TRO452 and TRO453/TRO447.

pGi55 was designed to allow ectopic integration of the *hfpY* gene under the control of P*_hfpR_* promoter and was constructed by Gibson assembly of a plasmid fragment carrying the P*_hfpR_* amplified using pGi44 DNA matrix and primers TRO519/TRO520 and the *hfpY* coding sequence fragment amplified using OSU THCO2-90 chromosomal DNA matrix and primers TRO521/TRO522.

Supplementary Figures

F1. Expression profiles of TonB-dependent receptors in *F. psychrophilum* OSU THCO2-90.

Quantile-normalized log2-expression level of mRNAs encoding HfpR (top) and BfpR (bottom) across 32 biological conditions in duplicates as previously published in Guérin *et al* [2]. A full description of the biological conditions used in the condition-dependent transcriptome dataset is available at <https://fpeb.migale.inrae.fr/>. Briefly, iron deprivation (DIP) was generated by TYES broth supplementation with 25 µM 2,2’-dipyridyl. Plasma exposure was performed by incubating bacterial cells with 50% rainbow trout fish plasma during 5 min (Plasma). Blood conditions correspond to bacterial colonies grown either on TYES agar supplemented with 10% defibrinated horse blood (TYESA-Blood) or on 10% defibrinated horse blood agar only (Blood). Starvation conditions correspond to late stationary phase (T5) or colonies grown on TYES agar supplemented or not with fetal calf serum (TYESA-FCS; TYESA).

**
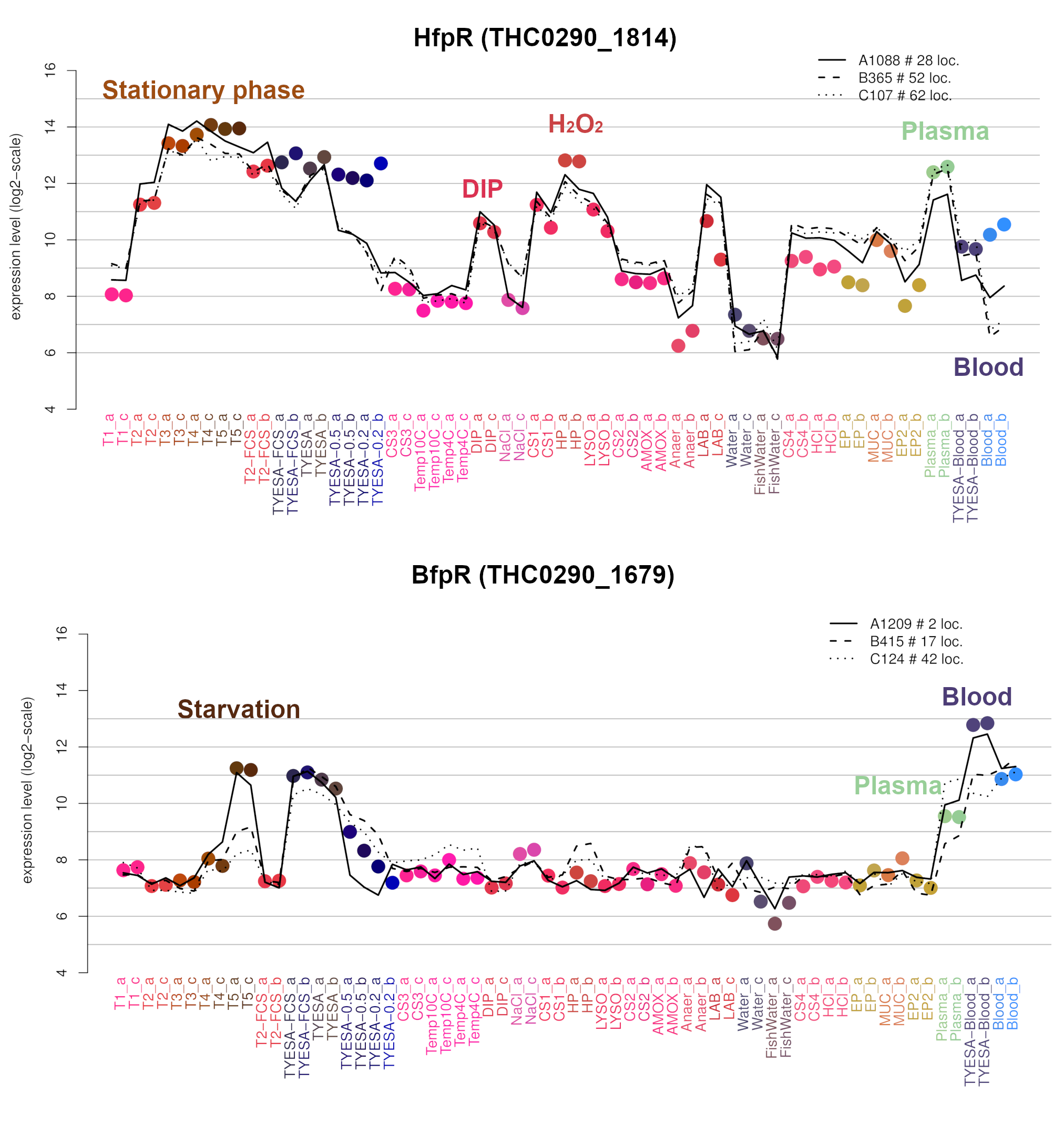
**

F2. Schematic representation of the *F. psychrophilum* chromosomal gene expression platform and set of plasmids used for reporter genes expression and mutant complementation.

The insertion site is within an intergenic region chosen for the absence of transcriptional signals and for its conservation within the *F. psychrophilum* species. pGi39 was constructed using pYT313 [1] as a backbone that carries, between homologous recombination regions, a chromosomal expression platform composed of a constitutive promoter (P*_rpsL_*), a multiple cloning site and an intrinsic terminator. Gene inserted into the MCS of pGi39 is under the control of the constitutive promoter of *rpsL*. Restriction sites available in the multiple cloning site of pGi39 are: NotI, SpeI, SacI, SalI, XmaI (nucleotide sequence: gcggccgccgactagtgagctcgtcgacccggg). Excision of the DNA fragment carrying the P*_rpsL_*, MCS and the intrinsic terminator can be achieved by double digestion of pGi39 with BamHI/PstI and allow cloning of a gene under its native expression signals using BamHI/PstI. Ectopic complementation of *hfpR*, *hfpY* and *bfpR* deletion mutants was performed by constructing a set of pGi39-derivative plasmids that allow gene insertion under the control of native promoter into the chromosome. Plasmids were constructed by Gibson assembly as described in **Methods M2**.


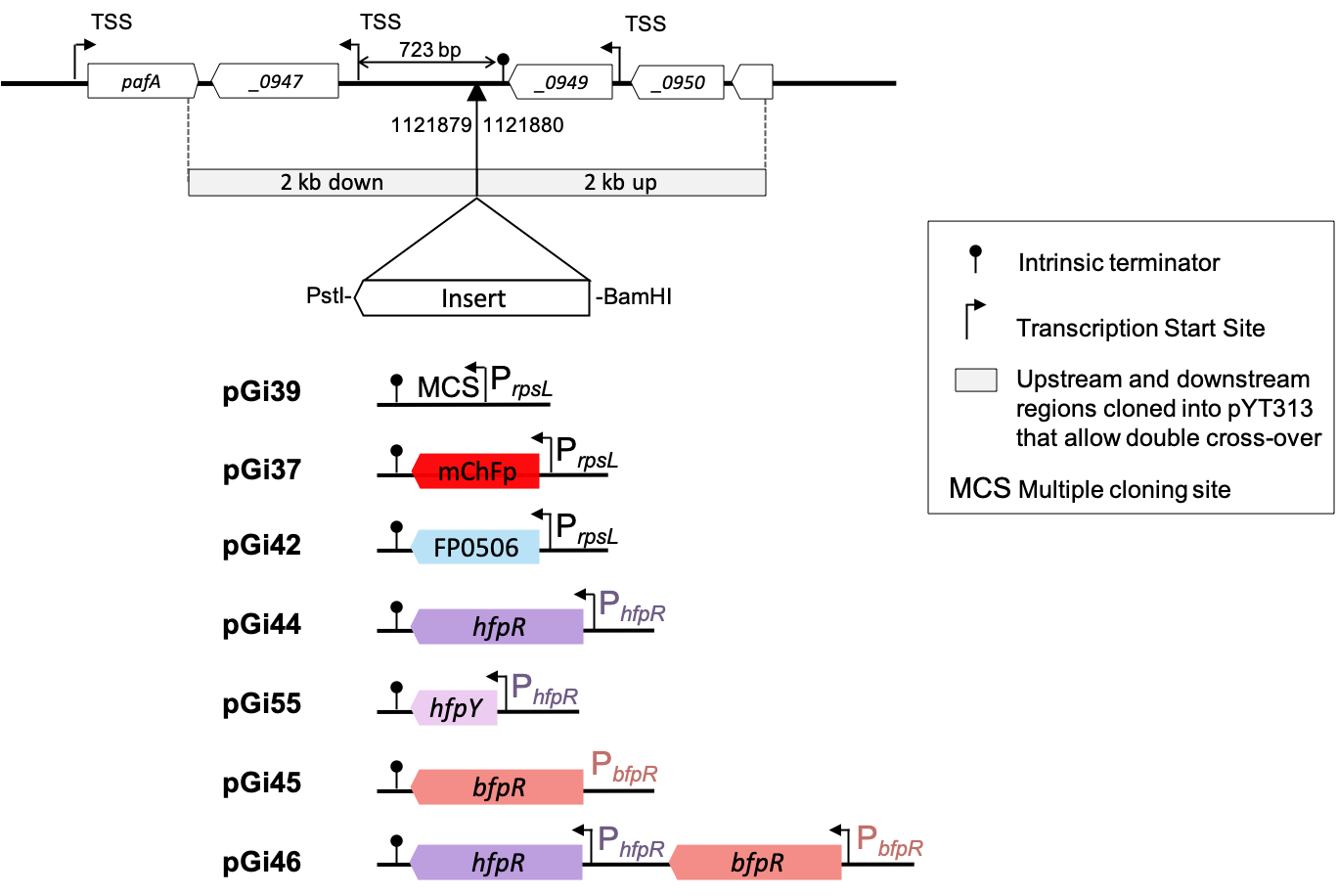


F3. Validation of the chromosomal expression platform using reporter genes in strain OSU THCO2-90.

**A.** mCherry expression of the strain carrying the P*_rpsL_*-mChFp construct inserted into the chromosome after recombination with pGi37 plasmid. Expression of the *mCherry* gene was monitored in stationary phase cultures performed in TYES broth using whole-cell fluorescence with a Tecan Microplate Reader (Infinite 200 PRO). Excitation and emission wavelengths were set at 535 nm and 610 nm, respectively. mCherry expression (in arbitrary units) was estimated by dividing fluorescence intensity by OD_600_. The mCherry expression was weak and near the background signal estimated using the wild-type strain, especially in the stationary phase. **B.** Elastinolytic activity of strain OSU THCO2-90 carrying the elastase-encoding gene FP0506 (from strain JIP 02/86)[3] at the chromosomal expression platform. Ten µL of stationary phase cultures performed were spotted on TYES agar supplemented with elastin (0.75%). Proteolytic activity was visualized as clearing zones around the bacterial growth after incubation at 18°C for 6 days. WT: OSU THCO2-90 wild-type; ecto::P*_rpsL_*-mCh: insertion of pGi37 by homologous recombination at the insertion site (between positions 1121879 and 1121880); ecto::FP0506: insertion of pGi42 at the insertion site.


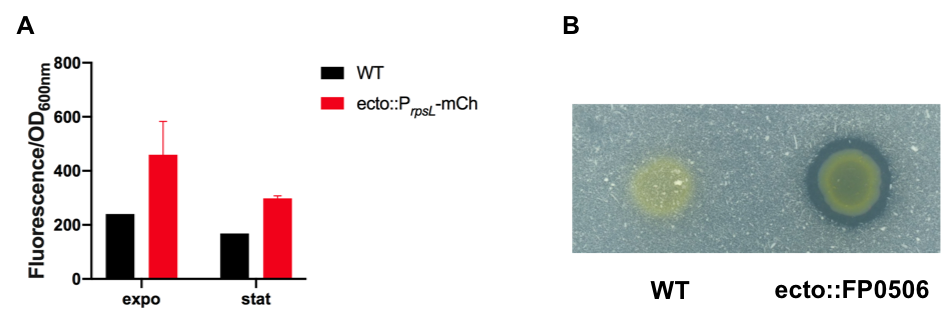


F4. Transcriptional structure of *hfp* and *bfpR* loci analyzed by circular 3’-5′ RACE experiments.

**(A)** Electrophoresis of amplicons from the first PCR (left) and the nested PCR (right) reactions of 3’-5′ RACE experiments. Total RNA treated by the pyrophosphohydrolase RppH (+RppH) or not (-RppH) was circularized, first-strand cDNA was synthetized using a primer specific of *bfpR* (TRO814), *hfpR* (TRO806) and *hfpY* (TRO811), then 3’-5’ junctions were amplified using a set of primers, as follows: for *bfpR* cDNA, with internal *bfpR* primers (a/d, 1^st^ PCR: TRO815/TRO816; nested PCR: TRO817/TRO818), *bfpR*-THC0290_1678 primers (b/d, 1^st^ PCR: TRO815/TRO825; nested PCR: TRO817/TRO826) or *asd*-*bfpR* primers (c/d, 1^st^ PCR: TRO815/TRO827; nested PCR: TRO817/TRO828); for *hfpR* cDNA with primers f/g (1^st^ PCR: TRO807/TRO808; nested PCR: TRO809/TRO810) and for *hfpY* cDNA with primers e/g (1^st^ PCR: TRO807/TRO812; nested PCR: TRO809/TRO813). **(B)** Determination of 5’ and 3’ ends of *bfpR* transcripts by TA-cloning and Sanger DNA sequencing of PCR products from +RppH treated samples. Boundaries of *bfpR* RNAs were identified by cloning 1^st^ PCR products generated using a/d and nested PCR products generated using b/d (no amplification was obtained with c/d). 1, 2 and 3 indicate the most frequent positions identified for 5’ends and 3’ end of *bfpR* mRNAs. **(C)** Determination of 5’ and 3’ ends of *hfpR and hfpY* transcripts by TA-cloning and DNA Sanger sequencing of PCR products from +RppH treated samples. Boundaries of *hfp* RNAs were identified by cloning 1^st^ PCR products generated using f/g and e/g. 1, 2 and 3 indicate the most frequent positions identified for 5’ends and 3’ end of *hfpR and hfpY* mRNAs. x-axis, genomic position in base pairs (bp) relative to the start position of downstream gene; y-axis, number of sequenced clones representative of each positions. Arrows indicate primers positions.

**
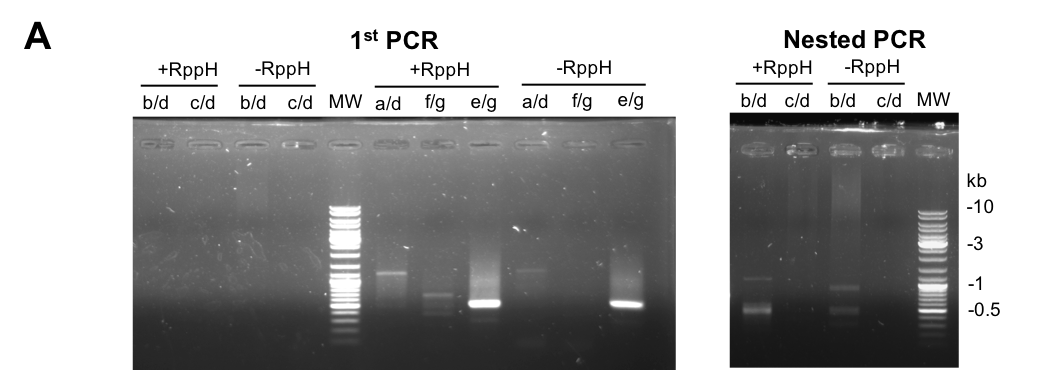
**

**
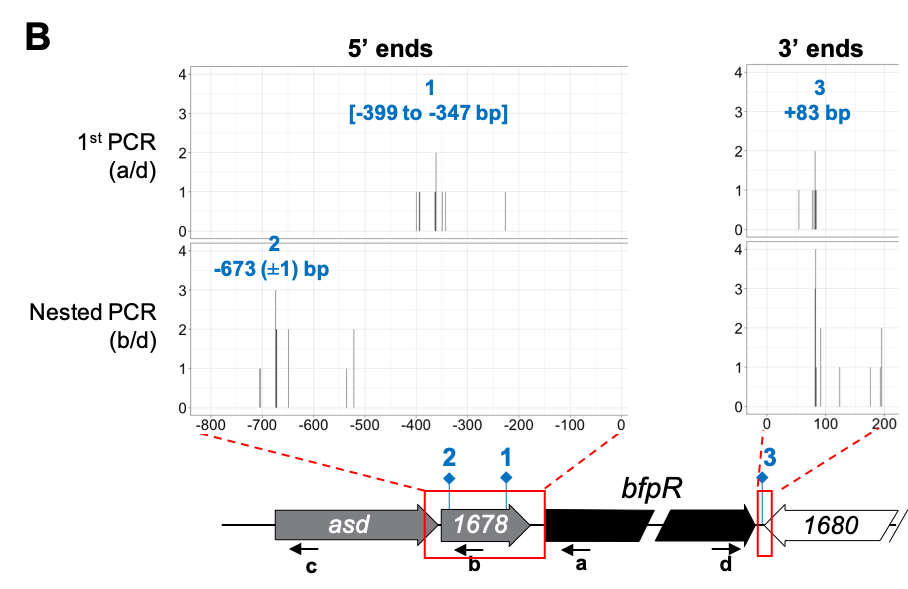
**

**
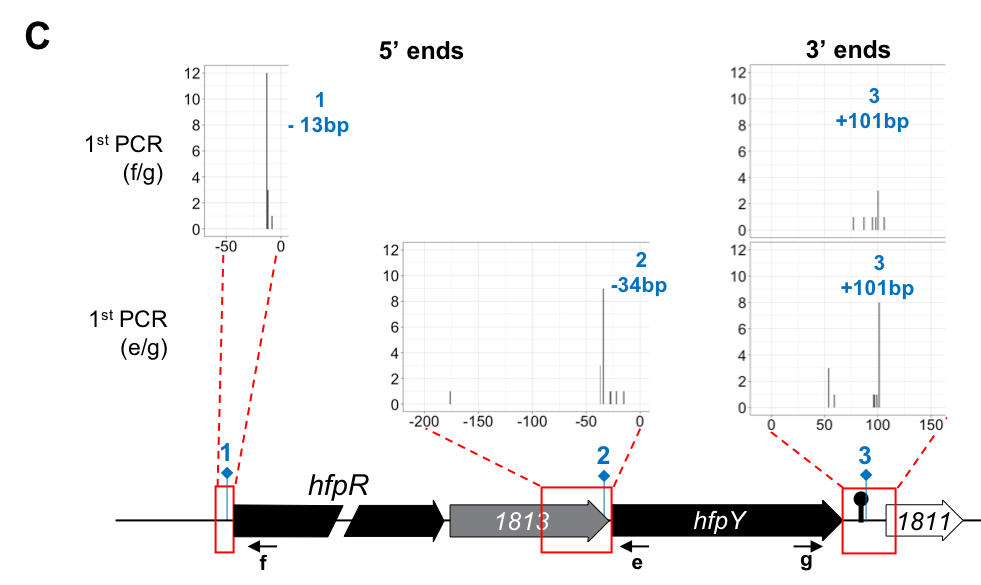
**

F5. Growth of *F. psychrophilum* OSU THCO2-90 at high concentration of hemoglobin.

Growth of the wild-type strain at high hemoglobin concentration determined by viable cell measurements (CFU/mL) in TYES broth supplemented with 1% hemoglobin (purple) or not (black). Values represent the mean and standard deviation of three (A-C) or six (D) independent experiments.

**
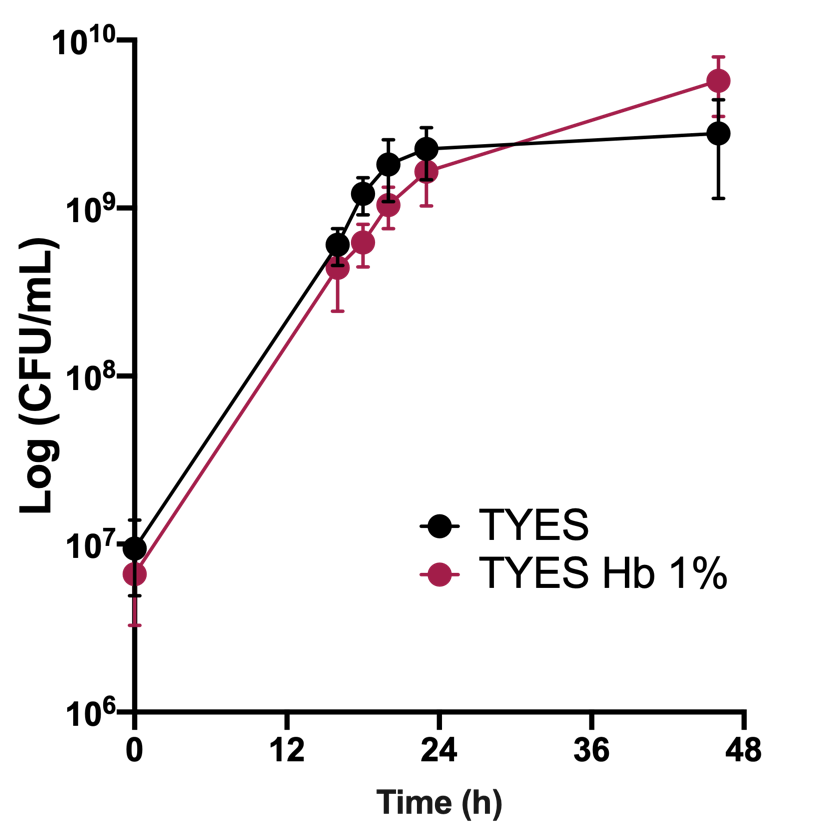
**

F6. RT-qPCR measurement of gene expression in complemented deletion mutants compared to wild-type.

mRNA level was quantified using RNA extracted from cultures of wild-type and deletion mutant carrying the corresponding ectopic gene copy (Figure F2). Cultures were performed in the biological condition corresponding to gene overexpression: TYES E20 Hb 1.25 µM for ecto::*hfpR* and ecto::*hfpY* strains, TYES Hb 1% for ecto::*bfpR* strain. Ct values of genes were normalized using the geomean of two reference genes (*rpsA* and *frr)*. RQ: Relative quantification of mRNA was expressed as 2^–∆∆Ct^ using wild-type as reference sample. Values are mean and standard deviation of three independent experiments. RT-qPCR showed that gene expression was restored in all complemented strains. Deletion of *hfpR* did not affect the mRNA level of *hfpY* and ectopic complementation resulted in similar *hfpR* mRNA level relative to wild-type. Deletion of *hfpY* did not affect the mRNA level of *hfpR*, however ectopic complementation resulted in lower *hfpY* mRNA level relative to wild-type (17%). Ectopic complementation of *bfpR* restored only partially the mRNA level of *bfpR* compared to wild-type (13%).


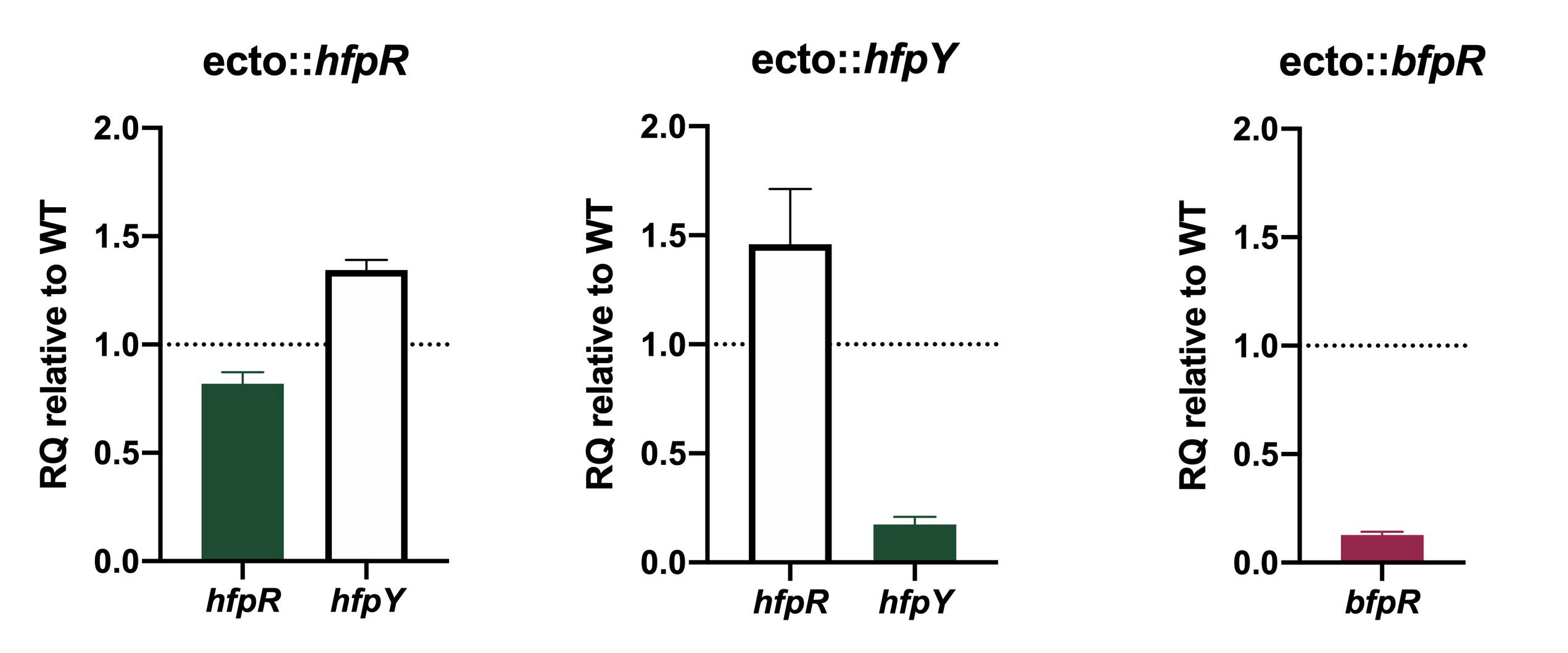


F7. Distinct *hfpR* and *hfpY* mRNA levels across several biological conditions assessed by microarrays.

Quantile-normalized log2-expression level of microarray probes targeting *hfpR* or *hfpY* across several biological conditions. Hybridization values of 15 *hfpR*- and 11 *hfpY*-specific probes were retrieved from Guérin *et al* [2]. Total RNAs were extracted from cells from log to stationary phases (T1 to T5), under iron-depletion (DIP), peroxide stress (H_2_O_2_), oxygen limitation (Hypoxia), exposure to rainbow trout plasma (Plasma), and from colonies grown on TYES blood agar or blood agar. CS1, CS2 and CS3 correspond to reference conditions for H_2_O_2_, Hypoxia and DIP stress conditions, respectively.


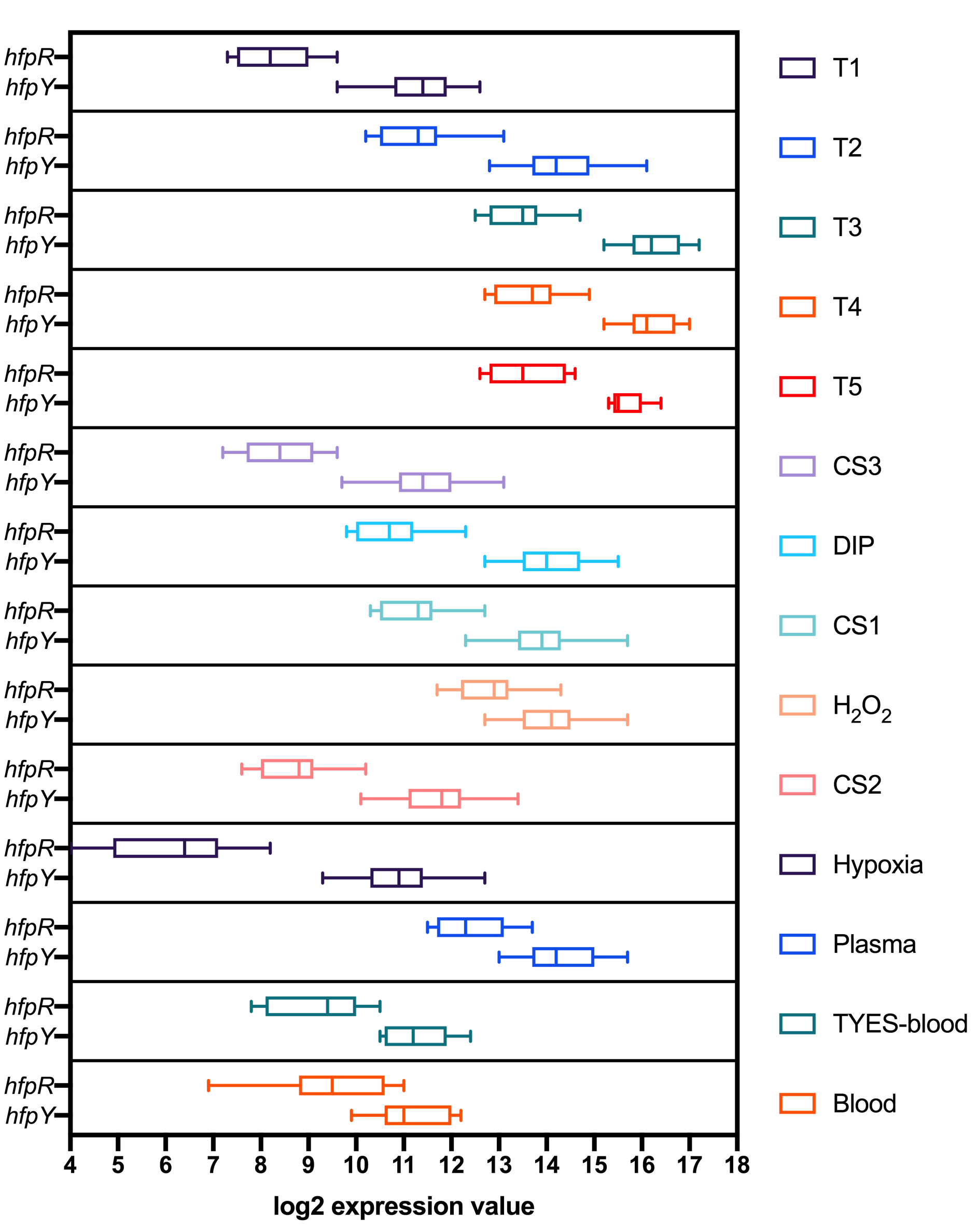


F8. THC0290_1813 encoding a predicted inner membrane protein of unknown function is not required for growth on hemoglobin as an iron source.

The THC0290_1813 mutant produced as part of a Tn*4351* mutant library in strain OSU THCO2-90 was used. Growth was compared to wild-type and Δ*hfpY* by optical density measurement at 600nm in 20 µM EDDHA iron-depleted TYES broth supplemented with 1.25 µM hemoglobin (▲) or not (♦). A reduced growth rate was observed only for Δ*hfpY* indicating that THC0290_1813 is not required under these conditions and that the Tn*4351* insertion does not block the expression of *hfpY* downstream gene. Wild-type: black, Tn4351::*1813*: pink, Δ*hfpY*: blue. Values represent the mean and standard deviation of three independent experiments.


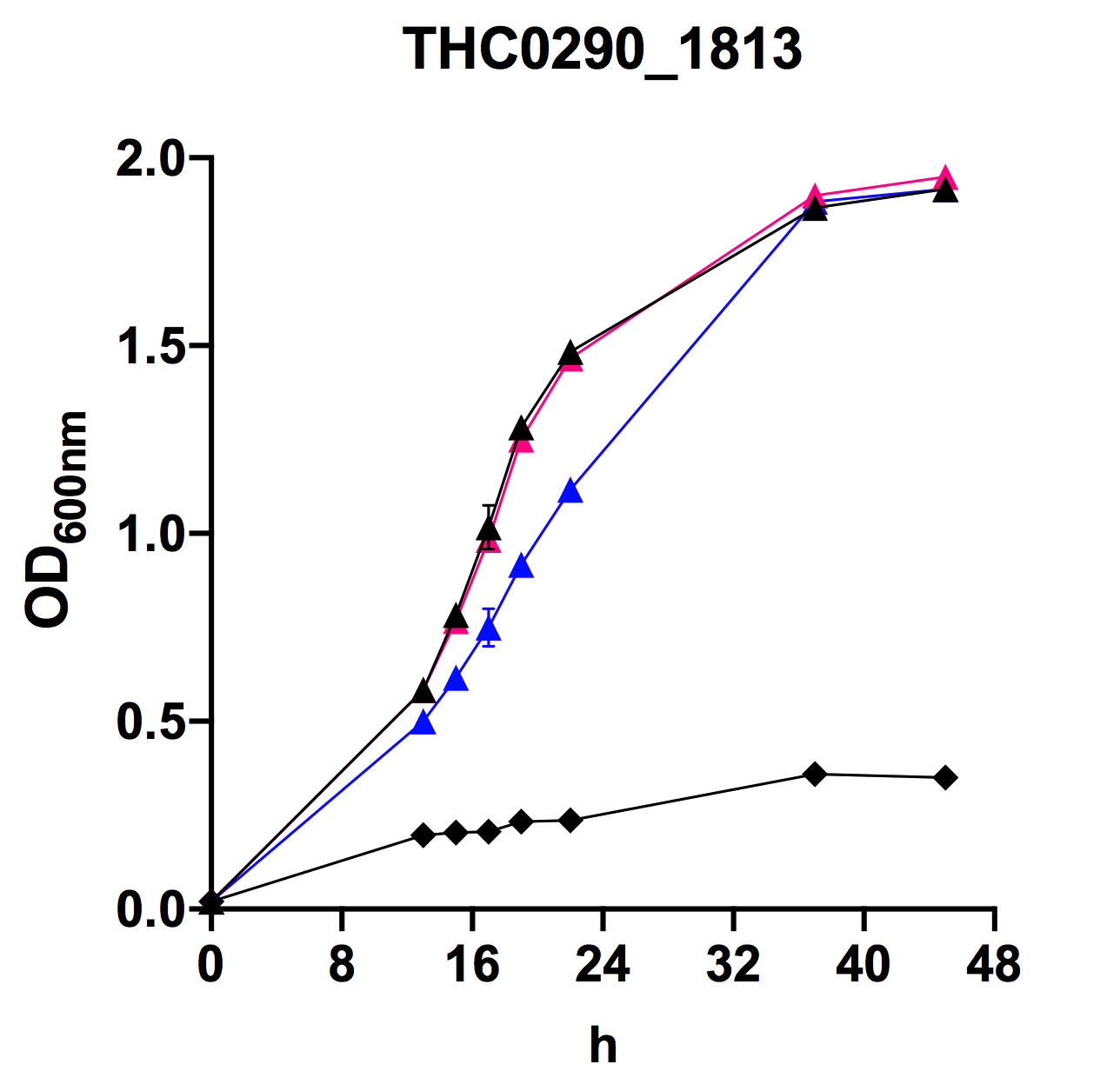


F9. MSA of HfpY-like protein of *F. psychrophilum* with 5 characterized HmuY homologs.

Multiple sequence alignment was constructed using PROMALS3D [4] with 5 sequences of HmuY-like proteins from *Porphyromonas gingivalis* (accession number: CAM31898), *Tannerella forsythia* (accession number: WP_046825712.1), *Prevotella intermedia* (accession numbers: AFJ07542, AFJ08449) and *Bacteroides vulgatus* (accession numbers: ABR39853) and the crystal structure of holo-HmuY from *Porphyromonas gingivalis* (PDB: 3H8T). Resulting alignment is provided below (*see* PROMALS3D web server for detailed output information). Sequences are colored according to predicted secondary structures (red: α-helix, blue: β-strand). consensus_ss: consensus secondary structure (α-helix: h; β-strand: e); consensus_aa: conserved amino acids are in bold and uppercase letters.

Conservation: 99 9 6 6 6

HmuY_Pg 1 MKKIIFSALCALPLI--VSLTSCGKK-----KDEPNQPSTP---------EAVTKTVTIDA--------S 46

3h8t_chainA_p001 1 --------------------------------------------------EAVTKTVTIDA--------S 12

Bvu_Bv 1 MKRY-LSII-TILGMMLLPFSACDGILEG-IYDSPAASDSNELGFIRTDPSTHSGTIYIDA--------T 59

Tfo_Tf 1 MKMRNVMTLALVALS--LAFVGC---------DKKDDVKET---------IKKS--KTIDA--------T 40

PinA_Pi 1 MKFKSFMAL-SCLTV--LLFSSCSNDDPT--PKPKPEQPKD---------VPTKTLSFITQEKAF----Q 52

PinO_Pi 1 MKTK-IFAV-ACLAT--LLFTSCSKDNN----DDPNPKPET---------TQVKHFETLM---------P 44

**HfpY_Fp** 1 MKNH-FFKVAVLALT--IFVSSCSKDEPATAVLTPQPQITT---------LEVKTVSNLNAPQVGMSFGP 58

Consensus_aa: **MK**...*h*.*hl*.*hhhhh*..*l*.*h*ot**C**.........cp.s...ps...........*h*pp*h*.*hl*s**A**........s

Consensus_ss: hhhhhhhhhhhhh hhhhh eeee

Conservation: 6 6 6 6 6 96 9 6 6

HmuY_Pg 47 KYETWQYFSFSKGEV--VNVTD--------------------------------YKNDLNWDMALHRYDV 82

3h8t_chainA_p001 13 KYETWQYFSFSKGEV--VNVTD--------------------------------YKNDLNWDMALHRYDV 48

Bvu_Bv 60 DYRRWTFIDFHTQKVDSVNVTD-----------------------------SEQ-KEPEEWDIAVHRYDV 99

Tfo_Tf 41 KYEMWTYVNLETGQT--ETHRDFSE--WHVMKN--------GKLLETIPAKGSEADIKIKWHIAIHRFDI 98

PinA_Pi 53 GYDKWVYVNLETGET--VMKDDVSEQEWRTYSDAGKKKDLFGKYDVTKTVEGKPSNAPDKWHLAFHVFDV 120

PinO_Pi 45 GYDSWIYIDLETGKF--EQQAELGKREFRKYKSM---MDPNYEVVGTEPAKGTDADLPKKWDIAFHITDA 109

**HfpY_Fp** 59 VSGEFTKFSFSENAV--VTN--------------------------------------DNWDVAFRGTTI 88

Consensus_aa: .**Y**cp**W***h@h*s*h*ppsp*h*...p.s**D**.................................c.s.p**W**c*l***A***h***H**.*h***D***l*

Consensus_ss: eeeee hh e ee ee eeeeeeeee

Conservation: 9 66 6 9 9 6

HmuY_Pg 83 RLNCGESGKGKGGAVFSG-----------KT---EMDQATTVPTDG-YTVDVL----GRITVKYEMGPDG 133

3h8t_chainA_p001 49 RLNCGESGKGKGGAVFSG-----------KT---EMDQATTVPTDG-YTVDVL----GRITVKYEMGPDG 99

Bvu_Bv 100 KTNAGAV-------LETG-----------FTGFSALRNADAMPEGA-YVEDVWTT--AKIAIDMSGMMDG 148

Tfo_Tf 99 RTNEGEA-------IATK-----------ET---EFSKVTGLPAGD-YKKDVEIKDKMLVGFNMADMMKS 146

PinA_Pi 121 RTNGAEA-------CMTD-----------TT---DIETIKTLPTNVKWVSDIK----AYLIYDMTGMMKN 165

PinO_Pi 110 RTNNGEV-------LMTG-----------ET---DLNKINALPAGN-YVADAP----ADIVVDMSRMQSE 153

**HfpY_Fp** 89 LVNGGAA-------IGIAGEPSRTGMGAVSIANNTLSGVTAFPAADTFRQDAN----SSYAIP-IGSGN- 145

Consensus_aa: **+***h***N**s**G**.*h*.......*l*.os...........p**T**...p*h*pp*h*s*hh***P***h*ss.*@*..**D***h*.....t.*lhh*s*h*....ps

Consensus_ss: ee ee ee hhhhhe eeee eeeee

Conservation: 6 9 96 96 69 9

HmuY_Pg 134 H-QMEYEEQGFSEVITGKKNAQGFASGGWLEFSHGPAGP-TYKLSK------RVFFVRGADGNIAKVQFT 195

3h8t_chainA_p001 100 H-QMEYEEQGFSEVITGKKNAQGFASGGWLEFSHGPAGP-TYKLSK------RVFFVRGADGNIAKVQFT 161

Bvu_Bv 149 N--IVYMESYYNEELS-----------KWLNVDKSNMPP-TYTLSN------KVYMVKLKDGTYAAVRLT 198

Tfo_Tf 147 K-FTVAGMAKVNPVLK-----------TWIVENPMGKAP-VLS--K------SVFVVKFKDGSYAKIKFT 195

PinA_Pi 166 PVVMGYMKSYVNMGLY-----------YWMHKVKGTMGEYALTMSKSDPKKAPVFLVKFKDGSYAVIQFT 224

PinO_Pi 154 G-VLGMVKTMLNGEMG-----------KWVKSN--GMGK-PKTVMG------NVFAVKFKNGNAALIKFK 202

**HfpY_Fp** 146 ---------------------------GWYNYN--GATN-IVTPLAG-----KVFVVKTHNGKYAKFEIL 180

Consensus_aa: ...*h*.*h*.cp.*h*s..*l*.............**W***h*p.s..s*h*s..*hh*o.........p**VF***h***VK***h***+**s**G**s*h***A**.*l*p*hh*

Consensus_ss: eeee ee eeeee eeeeeee

Conservation: 6 66 6 6

HmuY_Pg 196 DYQDA--------ELKKGVITFAYTYPVK----- 216

3h8t_chainA_p001 162 DYQDA--------ELKKGVITFTYTYPVK----- 182

Bvu_Bv 199 NYMNA--------SGVKGFMTIDYIYPFEL---- 220

Tfo_Tf 196 DATND--------KQEKGHVSFNYEFQPK----- 216

PinA_Pi 225 GLKDA--------TGKKKEVSFKYKFVKKN---- 246

PinO_Pi 203 DNLDK--------TGKKKAVSFDYKFIKKAK--- 225

**HfpY_Fp** 181 SYYKDTPANPDAATSVGRHYTFKFVYQANSTTSF 214

Consensus_aa: s*h*bss........p..**K**.*hh*o**F**p**Y**.*@*..c.....

Consensus_ss: eee eeeeeeeee

F10. Kaplan-Meier survival curves of rainbow trout after infection with wild-type, mutant and complemented strains.

Rainbow trout fingerling (Sy*Aut line, 5 g average weight) were infected by intramuscular injection. Each group (n=10) was challenged at a dose set to correspond to 1 LD50 (left column) or 10 x LD50 (right column) of the wild-type strain (black line). Exact viable cell counts were determined by plating bacterial suspensions in TYES agar and the results expressed as CFU/fish are indicated on the graphs. Comparison of wild-type and **(A)** Δ*hfpR* (red plain line) and ecto::*hfpR* (red dashed line); **(B)** Δ*hfpY* (blue plain line) and ecto::*hfpY* (blue dashed line); **(C)** Δ*bfpR* (green plain line) and ecto::*bfpR* (green dashed line); **(D)** Δ*bfpR*Δ*hfpR* (brown plain line) and ecto::*bfpR-hfpR* (brown dashed line). Survival curves were compared between groups of fish infected with mutant and wild-type using the Mantel-Cox log-rank test and the corresponding pvalue is indicated.


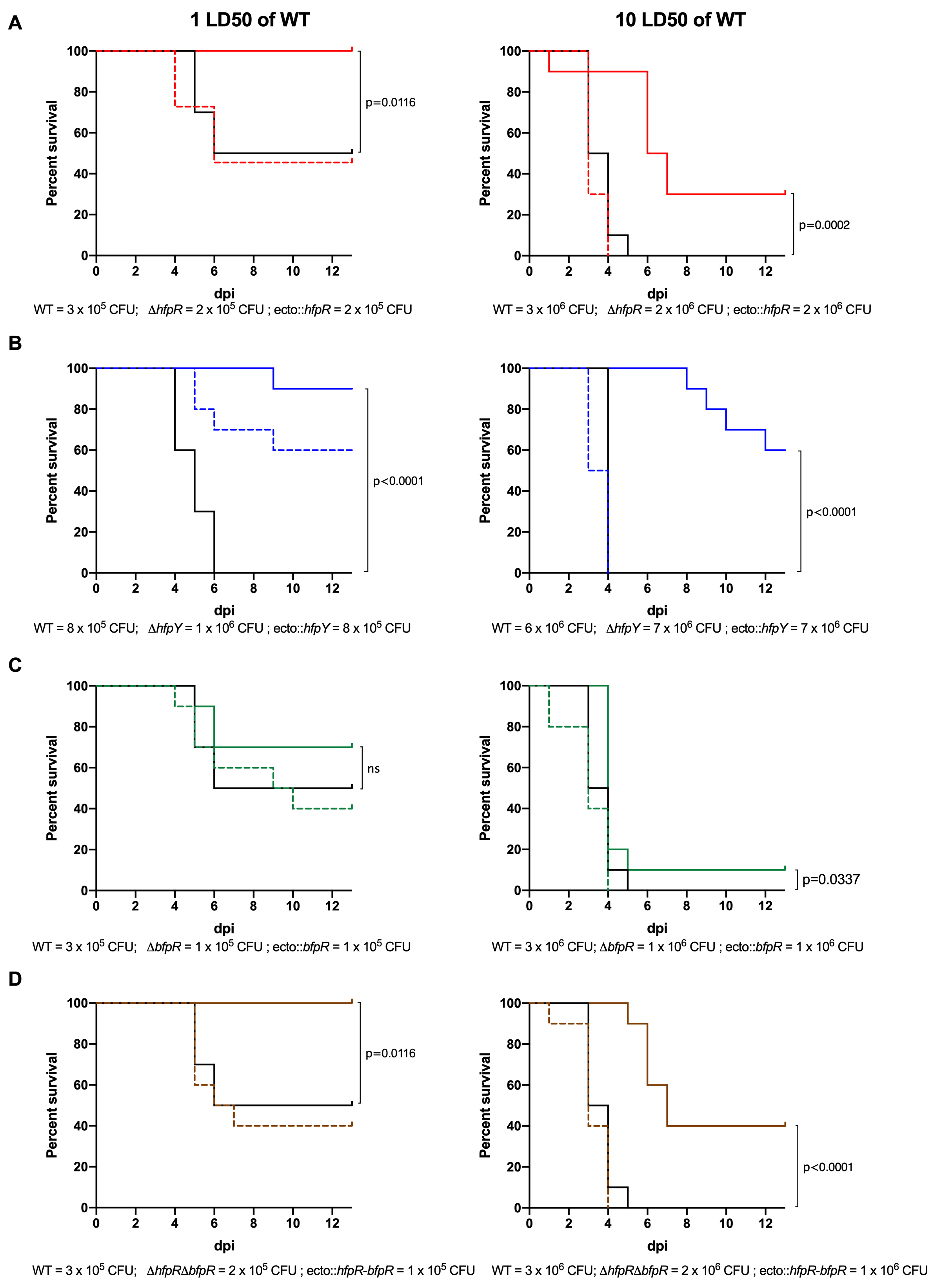


F11. MSA of HfpR and BfpR with other hemoglogin/heme TBDRs

Multiple sequence alignment was constructed using PROMALS3D [4] with 6 sequences of hemoglobin/heme TBDRs from Gram-negative bacteria and the crystal structure of ShuA from *Shigella dysenteriae* (PDB: 3fhh.1). Resulting alignment is provided below (*see* PROMALS3D web server for detailed output information). Sequences are colored according to predicted secondary structures (red: α-helix, blue: β-strand). consensus_ss: consensus secondary structure (α-helix: h; β-strand: e); consensus_aa: conserved amino acids are in bold and uppercase letters. Domains: predicted protein family domains retrieved from the MicroScope annotation platform (Carboxypeptidase-like, regulatory domain: IPR008969; TonB-dependent receptor Plug domain: IPR012910; β-barrel domain: IPR000531). The predicted 22 transmembrane segments of the β-barrel are underlined and the surface-exposed loops are numbered L1 to L11. Alignment results for *F. psychrophilum* BfpR and HfpR **(A)** and for THC0290_0681 and THC0290_0369 protein sequence **(B)**.

**A.** BfpR and HfpR display conserved residues within the surface-exposed loop L7.

Conservation: 9 5 7

ShuA_Sd 1 MSRPQFTSLRLSLLALAVSATLPTF--------------------------------------------------------------------------- 25

3fhh_chainA_p001 ----------------------------------------------------------------------------------------------------

PhuR_Pa 1 MPLSPP--FALRPC-LALLLLSPSLALAG----------------------------------------------------------------------- 26

HutA_Vc 1 MY-------KKSLLSSAIMLALVPSAYAD----------------------------------------------------------------------- 22

HpuB_Nm 1 MPIPF----KPVLAVAAIAQAFPAF-AADPAP-------------------------------------------------------------------- 27

HgbA_Hd 1 MKAN-----KLSAITLCILGYAHTV-YAESNM-------------------------------------------------------------------- 26

HmuR_Pg 1 MKSVVT---KQALIGLLFFSISIYSHAANPPAQPT----------------------------------------------------------------- 32

**HfpR_Fp** 1 MVI------NKAFGLHLLLLSFVGF--SQE-KQL------------------------------------------------------------------ 25

**BfpR_Fp** 1 MK--------SFLVALLLGFAIFCN--AQN-KITGAITNSENKILETVLVSIPELNKETISDSNGNFLFLDLPKGKFKMSFSLVGYDEKLVSVFINKAEI 89

Consensus_aa: **M**........p..*hh*s*hhl*..*h*..s...**A**p.......................................................................

Consensus_ss: hhhhhhhhhhhhhhhh

Domains  **|** **Signal Peptide | | Carboxypeptidase-like, regulatory domain (IPR008969) .**

Conservation: 5 565 5 5 5 5 595 5 5 79 5 55 579 5 6

ShuA_Sd 26 ---------AFATETMTVTATGNARSSFEAPMMVSVIDTSAPENQTATSATDLLRHV-PGITLDGTGR-TNGQDINMRGYDHRGVLVLVDGIRQGTDTG- 113

3fhh_chainA_p001 1 ------------TETMTVTATGNARSSFEAPMMVSVIDTSAPENQTATSATDLLRHV-PGITLDGTGR-TNGQDINMRGYDHRGVLVLVDGIRQGTDTG- 85

PhuR_Pa 27 --------NAVPLTPTTITATRTEQAVDSVPSTVSVQTREQLDRQNVNNIKELVRYE-PGVSVGGAGQRAGITGYNIRGIDGNRILTQIDGVELPNDFFS 117

HutA_Vc 23 --------DYASFDEVVVSTTRLNTQITDSAASVAVINASDIEQQMAEDIEGLFKYT-PGVTLTTNSR-QGVQGINIRGIEGNRIKVIVDGVAQPNQFDS 112

HpuB_Nm 28 -------QSAQTLNEITVTGTHKT-----QKLGEEKIRRKTLDKLLVNDEHDLVRYD-PGISVVEGGR-AGSNGFTIRGVDKDRVAINVDGLAQAESRSS 113

HgbA_Hd 27 --------QTEKLETIVVSSEDDS--VHNKNVGEIKKNAKALSKQQVQDSRDLVRYE-TGVTVVEKGR-FGSSGYAIRGVDENRVAVVVDGLHQAETISS 114

HmuR_Pg 33 ---DTIVSGNIALEDIVVTGSRTARLLKDVPVPTKVFKAKDIKAIAPSSFIDVLQYILPGIEFTKHGS---RDQLNAQGFDESSILFLVDGELISTGSTS 126

**HfpR_Fp** 26 ----SDSVKVATLNDVVITATRTERQLSSLPLPISIITSADISKAGISRLNEILTEQ-TGLITVPDFG--GAEGIQVQGLDAAYTLILIDGVPLVGRSAG 118

**BfpR_Fp** 90 NLNIRLESSSNHLEEVKIAATIFNKIQSQNVMKIEHKSIRELQQKGAATLMEGLASI-AGVSQVSTGT--SIGKPVIRGLSGNRVLVYTQGVRLENQQFG 186

Consensus_aa: ........ps..*h*pp*hhl*oto...p..pp.s*h*....bp.p.*l*ppb.*h*ps..**-***ll*p...s**G***l*p*h*s..tp..t.p.*h*s*h*p**G***h***-**.s.*lhh*.*l***DG***l*.bspp..t

Consensus_ss: eeeeee hhh eeeee hhhhhh hhhhhhh eeee eeeeeee eeeee ee

Domains  **| TonB-dependent receptor Plug domain .**

Conservation: 557 696 7 5 99 7679 5 5 5 7 5 5 5 5 5

ShuA_Sd 114 -------------HLNGTFLDPALIKRVEIVRGPSALLYGSGALGGVISYDTVDAKDLLQEGQSSGFRVFGTGGTGDHSLGLGASAFGRTENLDGIVAWS 200

3fhh_chainA_p001 86 -------------HLNGTFLDPALIKRVEIVRGPSALLYGSGALGGVISYDTVDAKDLLQEGQSSGFRVFGTGGTGDHSLGLGASAFGRTENLDGIVAWS 172

PhuR_Pa 118 GP---------YAQTHRNYVDPDIVKRVEILRGPASALYGSNAIGGAVSYFTLDPSDIIKDGKDVGARLKAGYESASHSWLTSATVAGRADDFDGLLHYG 208

HutA_Vc 113 GN--------SFLNSSRVDIDTDMVKSVEIVKGAASSLQGSDAIGGIVAFETKDPADILK-GRNMGGYAKLNYSSSDKTFSESIALANKSGDLESLVAYT 203

HpuB_Nm 114 EAFQELFGAYGNFNANRNTSEPENFSEVTITKGADSLKSGSGALGGAVNYQTKSASDYVSEDKPYHLGIKGGSVGKNSQKFSSITAAGRLFGLDALLVYT 213

HgbA_Hd 115 QGFKELFEGYGNFNNTRNGVEVENLKQAVIQKGADAIRTGSGSLGGTVSFESKDARDYLI-DKNYHFGYKTGYSSADNQKLHSVTAAGRYSDFDLLAVHT 213

HmuR_Pg 127 -------------GIDFERINPDDIERIEVLRGASSALYGSNAIGGVINIITRTAK------DPFRVSASARYDSRD-GQKYDVAAGVKRGIFTSQSGVQ 206

**HfpR_Fp** 119 -------------TLDLSRISVGNIDRIEIVKGASSSLYGSEALAGVINIITKKPK-----SDHFSGNLSYRYASFN-THDATANFVWKKKKFSGSFFTN 199

**BfpR_Fp** 187 -------------EEHGLGLNESGIESVEVIKGPASLLYGSDALGGVLYFNPEKFAK----PNSYLADFSQKYFSNTNGSNTSFGLKSSSENWKFLIRGA 269

Consensus_aa: .............p.p...*l*ps..*l*cp*l*p*lh***+G**sst.b.**GS**s**A***l***GG***hl*s*h*.o.ps.**D**....sps*h*...*h*p....o.sp....s*h*s*h*.s**+**...*h*p.b*h*.*h*s

Consensus_ss: hhhh eeeeee eeeeeee eeeeeeeeeeee eeeeeeeee eeeeeeee

Domains **| β-Barrel Domain L1 .**

Conservation: 5 7 5

ShuA_Sd 201 SRDRGDLRQSNGE-----------------------------------TAPNDESINNMLAKGTWQIDSAQSLSGLVRYYNNDAREPKNPQTVEAS---- 261

3fhh_chainA_p001 173 SRDRGDLRQSNGE-----------------------------------TAPNDESINNMLAKGTWQIDSAQSLSGLVRYYNNDAREPKNPQTVEAS---- 233

PhuR_Pa 209 YRQGHETESNGGHGG--------------------------TGLSRSEANPEDADSYSLLGKLGWNYAEGSRFGLVFEKYKSDVDTDQKSAYGGPYDKGK 282

HutA_Vc 204 RRDGQEIQNFGSP------------------------------------DQQDNNANNLLVKLQYQLNPKHRLEFSGNYIRNKNDLENLEFS-------- 259

HpuB_Nm 214 RRFGKETKNRSTEGNVEIKNDGYVYNPTDTGGPSKYLTYVATGVARSQPDPQEWVNKSTLFKLGYNFNDQNRIGWIFEDSRTDRFTNELSNLWTGTTTS- 312

HgbA_Hd 214 QRHGNELRNYGYRH----------------------YDGSVVRKEREKADPYKITKQSSLIKIGYQLNDTNRFTLGYDDSRNTSRGTDWSNAFTSYNGG- 290

HmuR_Pg 207 YRADKSYILADQFE--------------------------------QELNVAGNTTWNINQKFTISPTENLSFNLTGLVNLRKQHWTD------------ 262

**HfpR_Fp** 200 YFDTNGYDLDKKNS---------------------------------LQTVEPYYNTTIQPKLYYDFSDKLKLIVGTRFYHQKQDNKTIINNK------- 259

**BfpR_Fp** 270 YNNHGDYKMPSDSL----------------------------------VTNTRFEEADVKTALGFSNS-KFSSVLRYNLNHLNLGIPEQIGLQTT----- 329

Consensus_aa: .p...p*h*p..s......................................ss......s*h*b.**K***h*.*@*p.sp..p*h*.*h*..p..ppp....pb...........

Consensus_ss: eee ee eeeeeeeeeeeee eeeeeeeeeee

Domains **L2 L3 .**

Conservation:

ShuA_Sd 262 -----------ESSNPMVDRSTIQRDAQLSYKLAPQGNDWLNADAKIYWSEVRINAQNTGS--------------------------------------- 311

3fhh_chainA_p001 234 -----------ESSNPMVDRSTIQRDAQLSYKLAPQGNDWLNADAKIYWSEVRINAQ------------------------------------------- 279

PhuR_Pa 283 PAIPPSMLPGGMYQWRKGNDTLTRERYGLEHHFLLDSQVADRIQWSLNYQLAKTDQATREFYYPIT---------------------------------- 348

HutA_Vc 260 -----------GYKNASGTDETTQYQLGIKHIWDAEFSLADRITWQFDVVGKEETGITDRTSKS------------------------------------ 312

HpuB_Nm 313 ---------AATGDYRHRQDVSYRRRSGVEYKNELEHGPWDSLKLRYDKQRIDMNTWTWDIPKNY----------------------------------- 368

HgbA_Hd 291 ---------PFLKDVRHTNDQSNRKNISFVYENFDTNDFWDTLKITHNHQKIKLKARLDEYCDVNGEIDCPAIANPSGLYINDKGIFLDKHDGEITHKKE 381

HmuR_Pg 263 ----------------KIDFLYNSYDVKAGANWRISETSDLDISYHYDKYSRDTCLIKTEN--------------------------------------- 307

**HfpR_Fp** 260 --------------NYQGDAVINEWNSQIKLEHNWNSK--INSEYEIYLTNYKTDAFLNNSK-------------------------------------- 305

**BfpR_Fp** 330 -----------KRDPDYPRQGVVNHILSLHNNFYFRN---SKFDADFGYISNSRKEFADSTI-------------------------------------- 377

Consensus_aa: .............p....p....p.p..*h*.*h*p...p.p.*h*.p*h*p*h*p*h*.....c.p...pp........................................

Consensus_ss: eeeeeeeeeeeeee eeeeeeeeeeeeeeeeeee

Domains **L4 .**

Conservation:

ShuA_Sd ----------------------------------------------------------------------------------------------------

3fhh_chainA_p001 ----------------------------------------------------------------------------------------------------

PhuR_Pa 349 ------------------------------------------------------------------------------------------------RKVL 352

HutA_Vc 313 ------------------------------------------------------------------------------------------------NGNI 316

HpuB_Nm 369 -------------------------------------------------------------------------------------------DLRGINSEV 377

HgbA_Hd 382 GEFNNYFDSKGKEVRVKGFNVDSILINCDQYDCSKPMQLLSSTNNGYGGSPNKYIYKTYELFEKTMNNGNGKYAVLEIRSSGHEKFSRVYLPSEKGYVEN 481

HmuR_Pg ----------------------------------------------------------------------------------------------------

**HfpR_Fp** 306 --------------------------------------------------------------------------------------------------NI 307

**BfpR_Fp** ----------------------------------------------------------------------------------------------------

Consensus_aa: ....................................................................................................

Consensus_ss:

Domains **.**

Conservation: 5 9 5

ShuA_Sd 312 --SGEYREQITKGARLENRSTLFADSFASHLLTYGGEYYRQEQHPGGAT--------------------------------------------TGFPQAK 365

3fhh_chainA_p001 280 ----EYREQITKGARLENRSTLFADSFASHLLTYGGEYYRQEQ--GSHT--------------------------------------------TGFPQAK 329

PhuR_Pa 353 RTRDTTYKERLWVFDSQLDKSFAI-GETEHLLSYGINLKHQKVTGMRSGTGTNLDT-------------------------GADSPRDALERSSDFPDPT 426

HutA_Vc 317 QKKDYLYSDKGFSFDSQLDKSFMV-SNTEHYIVYGFSLSDKDIENTNQEFNSI----------------------------------GKNNVIFYIPNAS 381

HpuB_Nm 378 YHSFRHIRQNTAQWTADFEKQLDF-SKAVWAAQYGLGGGRGDNANSDYSYFVKLY--------------------------DPKILTSNQAKITMLIENR 450

HgbA_Hd 482 QWKDRDLNTDTQQYNIDLTKSFKL-KSVEHNATYGGLYSEVKKSMTNRAGYEAYNRQWWANIFFGKENNKPNKCQPYNGNSFTTLCSHEDRLFSFLIPVK 580

HmuR_Pg 308 QKKLPIFDEDMHHLRAQYNLNLA----EVHLLNVGLEYIHDNVASPRLSSP------------------------------------------NDPGEKS 361

**HfpR_Fp** 308 LFEESFYDQWLIRPEIRTTFSI-----NNNKLTAGIGINHETLDRTYFGI-----------------------------------------------NAN 355

**BfpR_Fp** 378 --PVLDMNLNTLNYDLKYYLPNI----GKLEAIIGVQGMSQSNKNLGSEL--------------------------------------------LIPDAK 427

Consensus_aa: ..p...*h*pp.*h*..*h*p.p*h*pbs*h*.....s.*h*.*h*.*h***G**....c.p...................................................s.*h*.psp

Consensus_ss: eeeeeeeeeeeeeeeeeee eeeeeeeeeeeeeeeee e

Domains **L5 .**

Conservation: 5 5 5 7 959 5 5

ShuA_Sd 366 IDFSSGWLQDEITL-RDLPITLLGGTRYDSYRGSSDGY--------------------------------------------KDVDADKWSSRAGMTINP 420

3fhh_chainA_p001 330 IDFSSGWLQDEITL-RDLPITLLGGTRYDSYRGSSDGY--------------------------------------------KDVDADKWSSRAGMTINP 384

PhuR_Pa 427 VKTYALFAQDSISW---NDWTFTPGLRYDYTRMEPHITDEFLRTMK---------------------------QSQNTAVDESDKKWHRVSPKFGVTYDF 496

HutA_Vc 382 EKRYGFFIQDEIAF---DNLIVTPGIRFDSFETKPGDTSANPSL--------------------------------NDASEYKKYSDSALTARLGTVYKL 446

HpuB_Nm 451 SKYKFAYWNNVFHLGGNDRFRLNAGIRYDKNSSSAKDDPKYTTAI-----------------------------RGQIPHLGSERAHAGFSYGTGFDWRF 521

HgbA_Hd 581 TKTGALYVTDKIKL--NDKVNLDVAYRYDRIKHDPKYIPGTTPKLPTDLILGRFIEFKPKNTYATQDEKNENAEKNAVYLASKKTKFSANSYSATFSFDP 678

HmuR_Pg 362 VNNKILYGQYIYKV--TPKLVLSYGGRLDKHSGFG----------------------------------------------------LYYTSRLSAMYKC 407

**HfpR_Fp** 356 FNSQYGFLQYDYNP--SKKWNILAGFRYDNHNQYK----------------------------------------------------SQLSPKIAINYKV 401

**BfpR_Fp** 428 TNDIGALLTTNYEW---KSNVIQAGFRYDTRKINTESHGIIGDE---------------------------------GYFEAISKKYTSFNASVGYKTKL 491

Consensus_aa: .p....*@h*p..*h*p*h*....p*h*.*l*.s**G***h***R***@***D**p.p..sc.*h*............................................pc.p*h*..*h*o.p*h*t*h*.*h*c.

Consensus_ss: eeeeeeeeeeeeee eeeeeeeeeeeeeeee eeeeeeeeeeee

Domains **L6 .**

Conservation: 55 77759 5 5 5 6 7 9559 5

ShuA_Sd 421 TNWLMLFGSYAQAFRAPTMGEMYNDSKHFSIGRFYTNYWV--------------------------PNPNLRPETNETQEYGFGLRFDDLMLSNDALEFK 494

3fhh_chainA_p001 385 TNWLMLFGSYAQAFRAPTMGEMYNDSKHFSIGRFYTNYWV--------------------------PNPNLRPETNETQEYGFGLRFDDLMLSNDALEFK 458

PhuR_Pa 497 AQHYTWYGQYAQGFRTPTAKALYGRFENLQA----GYHIE--------------------------PNPNLKPEKSQSFETGLRGKFD-------EGSFG 559

HutA_Vc 447 NQENRLFAQISQGFRAPDFQELYYSFGNPAH----GYVFK--------------------------PNPNLEAEDSVSYELGWRYNAD-------SVSNE 509

HpuB_Nm 522 TKHLHLLAKYSTGFRAPTSDETWLLFPHP------DFYLK--------------------------ANPNLKAEKAKNWELGLAGSGK-------AGNFK 582

HgbA_Hd 679 MDFLKIQAKYATGFRAPTSDEIYFVFQHP------SFSIY--------------------------PNLYLKAERSKNKEVAITLHKQ-------KSFLT 739

HmuR_Pg 408 S-HVTNRLSYAEGYRAPSLQEMYFFFNHG------AFFIY--------------------------GNPDLKPEKSRMLSYSAEAHWN-------KLTLM 467

**HfpR_Fp** 402 TDHLSLKTAVGYGFKAPDFRQLYFDFTNSSV----GYTVLGYNVADTRLNALDNQGQILKRIKDISFDNPLKPESSLNFNLGIFYQRS-------KLKIE 490

**BfpR_Fp** 492 TDNLSFRLNVASGFRSPNLAELTSNGVHEGS----NRYEI--------------------------GNSNLKNEQNIQTDLNLEYGNS-------HFEFF 554

Consensus_aa: sp.*h*p*h*..p*h*tp**GFR**t**P**s..p*h@*....p.......s.*hh*b..........................s**N**ss**L+**s**E**ps.pbp*h*t*h*..p.p.........p*h*.

Consensus_ss: eeeeeeeeeeee hhhh ee eeeeeeeeeeeee eeee

Domains **L7 .**

Conservation: 5 9 5 6 7 5 5 9 7 5

ShuA_Sd 495 ASYFDTKAKDYISTTVDFA---------------------AATTMSYNVPNAKIWGWDVMTKYTTDLFS-------LDVAYNRTRGKDTDT--------- 557

3fhh_chainA_p001 459 ASYFDTKAKDYISTTVDFA---------------------AATTMSYNVPNAKIWGWDVMTKYTTDLFS-------LDVAYNRTRGKDTDT--------- 521

PhuR_Pa 560 VAVFYNKYRDFIDEDALNTDST---------------GGNGQTFQSNNIERAVIKGVELKGRLELGAFGA-PQGLYTQGSVAYAYGRNKDN--------- 634

HutA_Vc 510 LSIFYSDYDNFIDSQIVSGSF----------------KTRDAVHQSINIDKATIKGIELSNQFFWDRFMP-IVGFSSRIAAAYTEGKDGN---------- 582

HpuB_Nm 583 LSGFKTKYRDFIELTYMGVSSDNPNKPTYAPLSDGTALVSSPVWQNQNRSSAWVKGLEFNGTWNLDSIGL-PQGTHAGVNVSYIKGKAKQTNG------- 674

HgbA_Hd 740 VNLFQTDYKDFLDLAYLKKGSLP--------YGNGGSQLETLLYQNVNRDKARVKGLEVNSKLHLGDVWRTLDGFNLSYKLSLQKGRMSSKVGEEGKQRD 831

HmuR_Pg 468 GNVFFNHVRNRIDFTYKGT----------------------DLIYTNVSKEMRIFGFEGQANVVLPYGFG------FRASYSYTYDRWKATDKAG----- 534

**HfpR_Fp** 491 VNAFYNAISNLIDTRAIAIKY-----------------TGQNVFSYYNVGKIFTYGTEFNSAYSFSNNLK------ISLGYQYLIAKDKSVIDDFETHQY 567

**BfpR_Fp** 555 ANGFYNNISNYIFIAPNGSIM-----------------ADNDVYDYVQ-ANAKLYGGEFGIHFHPHPLDW------LHITSSFETVTGLKE--------- 621

Consensus_aa: *h*s.**F**bsp*h*ps*h***I**p..*h*........................s.*hh*...s..p*h*.*l*b**G***h***E***h*.sp*h*.*h*s..........*h*p*h*s*h*s*h*..s**+**..pp.........

Consensus_ss: eeeeeeeeeeeeeee eeeeeee eeeeeeeeeeeeee ee eeeeeeeeeeeeeee

Domains **L8 .**

Conservation: 5 5 5 65 55

ShuA_Sd 558 ----------------GEYISSINPDTVTSTLNIPIAHS----GFSVGWVGTFADRSTHISS--------------SYSKQPGYGVNDFYV--SYQGQQA 621

3fhh_chainA_p001 522 ----------------GEYISSINPDTVTSTLNIPIAHS----GFSVGWVGTFADRSTHIS-----------------SKQPGYGVNDFYV--SYQGQQA 582

PhuR_Pa 635 ----------------GEPINSVNPLTGVFGLGYDEADG----NYGGLLSWTLVKRKDRVDDSTFHTPD----GTASQFKTPGFGVLDLSA--YYRLS-- 706

HutA_Vc 583 ----------------GKPLNSVSPWNAVTGINYDSEN-----NWGTAVNLTYTAKKKASEIN------------GDYQPISSATVIDVTA--YYKPI-- 645

HpuB_Nm 675 ---------------QETPINALSPWSAVYNLGYDAPSK----RWGINAYATRTAAKKPSDTVHSNDDL--NNPWPYAKHSKAYTLFDLSA--YLNIG-- 749

HgbA_Hd 832 TNK------------LDTPMNAIQPQTHVVGVGYEHPQE----KFGVDMYLTHASAKKEKDTFNMFYDGKDQKDQHIKWRSDRYTLVDLIA--YVKPV-- 911

HmuR_Pg 535 ---------------TEMKLSNTRPHAATATLSYGHNFSKNY-RLASNFSTRFLSDLKTGRMNS--------NELFEEITYPGYTICRLDI--ENHFY-- 606

**HfpR_Fp** 568 IRDSQTLETIKINKVAYFGLFNRSKHTANVKFLYMIPKI----KTDINLRVFYRSKYGLFDTNNNQ------ILDVYDDFVKGYFITNVSI--SKQIK-- 653

**BfpR_Fp** 622 ---------------NGEYLPLIPANKWNNNIRTEFNLNNWIKKGFATLNIESIFKQNNNST--------------FETKSKDYTLVNVGLGGNININ-- 690

Consensus_aa: ...................*l*ss*h*ps.s*h*s.s*l*.*h*..s......p*h*s.s*h*.*h*p.*h*..bp..p.....................s*@*s*lh*s*l*.*h*....p....

Consensus_ss: eeeeeeeeeee eeeeeeeeeeeeeeeee eee eeeeeeee eeee

Domains **L9 L10 .**

Conservation: 5 5 9 6 5 79

ShuA_Sd 622 LK-GMTTTLVLGNAFDKEYWSPQGI-----------------------PQDGRNGKIFVSYQW 660

3fhh_chainA_p001 583 LK-GMTTTLVLGNAFDKEYWSPQGI-----------------------PQDGRNGKIFVSYQW 621

PhuR_Pa 707 -K-DLTLNAGLYNLTDKKYWLWDDVRGYDSVGEASALAP---ANIDRLSQPGRNFAVNLVWDI 764

HutA_Vc 646 -K-DLTLRAGVFNLTDEEYYNWNDVRGLPS-------------EDKDKTQAKRNFGITAKYEF 693

HpuB_Nm 750 -K-QVTLRAAAYNITNKQYYTWESLRSIREFGTVNRVDNKTHAGIQRFTSPGRSYNFTIEAKF 810

HgbA_Hd 912 -K-NVTLRAGVYNLTNREYGTWDSIRSIRPFGTTNLINQETGKGIKRFNAPGRNFRVNAEITF 972

HmuR_Pg 607 -R-NYTLHIGAENLFDYKPKALAFN---------------------SPTSPGRIIYGLVRITF 646

**HfpR_Fp** 654 -E-KFSFQVGANNLLDYTNPKEI------------------------SNLSGRQIFGKLQFNF 690

**BfpR_Fp** 691 -KLKFDISINANNMLNKSYISHLSRLK-----------------TDRISNIGRNIVLGVSFFL 735

Consensus_aa: .**+**.p*h*o*h*p*h*s*h*.**N***hh*sbp.....s........................s.s**GR**p*h*...*h*p*h*p*h*

Consensus_ss: eeeeeeeee eee eeeeeeeeee

Domains **L11 |**

**B.** The FRAP/NxxL motifs identified in loop L7 of heme/hemoglobin TBDRs are not conserved in THC0290_0681 and THC0290_0369 sequences.

Conservation: 5 666 8 6 6 9558

ShuA_Sd 415 GMTINPTNWLMLFGSYAQAFRAPTMGEMYNDSKHFSIGRFYTNYWVP--------------------------NPNLRPE 468

3fhh_chainA_p001 379 GMTINPTNWLMLFGSYAQAFRAPTMGEMYNDSKHFSIGRFYTNYWVP--------------------------NPNLRPE 432

PhuR_Pa 491 GVTYDFAQHYTWYGQYAQGFRTPTAKALYGRFENLQA----GYHIEP--------------------------NPNLKPE 540

HutA_Vc 441 GTVYKLNQENRLFAQISQGFRAPDFQELYYSFGNPAH----GYVFKP--------------------------NPNLEAE 490

HpuB_Nm 516 GFDWRFTKHLHLLAKYSTGFRAPTSDETWLLFPHP------DFYLKA--------------------------NPNLKAE 563

HgbA_Hd 673 TFSFDPMDFLKIQAKYATGFRAPTSDEIYFVFQHP------SFSIYP--------------------------NLYLKAE 720

**THC0290_0369** 657 NLKYGVSDKANIRFATSKTYTKPVIMEAFPLTYLNAD----GTSIQG--------------------------NSLLKNS 706

HmuR_Pg 402 SAMYKCS-HVTNRLSYAEGYRAPSLQEMYFFFNHG------AFFIYG--------------------------NPDLKPE 448

**HfpR_Fp** 396 AINYKVTDHLSLKTAVGYGFKAPDFRQLYFDFTNSSV----GYTVLGYNVADTRLNALDNQGQILKRIKDISFDNPLKPE 471

**BfpR_Fp** 486 GYKTKLTDNLSFRLNVASGFRSPNLAELTSNGVHEGS----NRYEIG--------------------------NSNLKNE 535

**THC0290_0681** 396 HARYNPWEGGVLRFSAGRGKRSANIFAENQQLFGSSR----SFNILESS----------------------GKIYGLNPE 449

Consensus_aa: s*h*p*h*p..p...*h*...*h*tpt*@***+**s**P**s*h*..*hh*............s..*h*bs..........................s..**L**cs**E**

Consensus_ss: eeeeee eeeeeeeeeeee hhhh ee e

Domains **L7 .**

Supplementary Tables

T1. Strains and plasmids.

| **Strain or plasmid** | **Description^(a)^** | **Source or reference** |
| --- | --- | --- |
| **Strains** |  |  |
| *E. coli* DH5α Z1 | Strain used for cloning | Expressys |
| *E. coli* MFD*pir* | Strain used for conjugation | [5] |
| *E. coli* Top10 | Strain used for heterologous protein expression | Invitrogen |
| *F. psychrophilum* strain  OSU THCO2-90 | Wild type | [6] |
| *F. psychrophilum* strain TRV311 | ∆*hfpR* in strain OSU THCO2-90 | This study |
| *F. psychrophilum* strain TRV347 | ∆*hfpY* in strain OSU THCO2-90 | This study |
| *F. psychrophilum* strain TRV405 | ∆*bfpR* in strain OSU THCO2-90 | This study |
| *F. psychrophilum* strain TRV403 | ∆*hfpR*∆*bfpR* in strain OSU THCO2-90 | This study |
| *F. psychrophilum* strain TRV452 | ∆*hfpR* ecto::*hfpR* in strain OSU THCO2-90 | This study |
| *F. psychrophilum* strain TRV458 | ∆*bfpR* ecto::*bfpR* in strain OSU THCO2-90 | This study |
| *F. psychrophilum* strain TRV459 | ∆*hfpR*∆*bfpR* ecto::*bfpR*-*hfpR* in strain OSU THCO2-90 | This study |
| *F. psychrophilum* strain TRV491 | ∆*hfpY* ecto::*hfpY* in strain OSU THCO2-90 | This study |
| *F. psychrophilum* strain TRV421 | ecto::P*_rpsL_-mChFp* in strain OSU THCO2-90 | This study |
| *F. psychrophilum* strain TRV422 | ecto::P*_rpsL_-*FP0506 in strain OSU THCO2-90 | This study |
| **Plasmids** |  |  |
| pCP*Gm*^r^ | *E. coli*–*F. psychrophilum* shuttle plasmid; ColE1 *ori* (pCP1 ori), Ap^r^ (Gm^r^) | [7] |
| pCP*Gm*^r^-P_less_-mCh (pGi47) | pCP*Gm*^r^ carrying a promoter less codon-adapted *mCherry* gene; Ap^r^ (Gm^r^) | [2] |
| pGi48 | pGi47 derivative with *mCherry* under the transcriptional control of *hfpR* promoter region (240 bp) | This study |
| pGi49 | pGi47 derivative with *mCherry* under the transcriptional control of *bfpR_1* promoter region (284 bp) | This study |
| pGi74 | pGi47 derivative with *mCherry* under the transcriptional control of *bfpR_2* promoter region (609 bp) | This study |
| pGi80 | pGi47 derivative with *mCherry* under the transcriptional control of *bfpR_3* promoter region (950 bp) | This study |
| pYT313 | Suicide vector carrying *sacB*; Ap^r^ (Em^r^) | [1] |
| pGi27 | pYT313 derivative used to delete *hfpR* | This study |
| pGi29 | pYT313 derivative used to delete *hfpY* | This study |
| pGi38 | pYT313 derivative used to delete *bfpR* | This study |
| pGi37 | pYT313 derivative used for chromosomal integration of P*_rpsL_*-mChFp reporter fusion at the neutral intergenic site (between 1121879 and 1124880 of OSU THCO2-90 genome) | This study |
| pGi39 | pYT313 derivative used for cloning gene into the chromosomal expression platform, carrying 2-kb fragments upstream and downstream of the neutral intergenic site and a K7 composed of P*_rpsL_*, a multiple cloning site (NotI, SpeI, SacI, SalI, XmaI) and an intrinsic terminator | This study |
| pGi42 | pGi39 derivative used for chromosomal integration of P*_rpsL_*-FP0506 | This study |
| pGi44 | pGi39 derivative used for chromosomal integration of P*_hfpR_*-*hfpR* | This study |
| pGi45 | pGi39 derivative used for chromosomal integration of P*_bfpR_1_*-*bfpR* | This study |
| pGi46 | pGi39 derivative used for chromosomal integration of P*_bfpR_1_*-*bfpR*-P*_hfpR_*-*hfpR* | This study |
| pGi55 | pGi39 derivative used for chromosomal integration of P*_hfpR_*-*hfpY* | This study |
| pMAL-c4X | Maltose binding protein (MBP) vector, P*tac* promoter, M13 *ori*, Ap^r^ | New England Biolabs |
| pMBP-HfpY | Expression of N-terminal MBP-tagged HfpY cloned into pMAL-c4X | This study |

**^(a)^** Antibiotic resistance markers: ampicillin, Ap^r^ is expressed in *E. coli* and erythromycin (Em^r^) and gentamicin (Gm^r^) in *F. psychrophilum*. The replication origin in parentheses is active in *F. psychrophilum*.

T2. List of oligonucleotides.

| **Name** | **Sequence 5’->3’** | **PCR Amplification** |
| --- | --- | --- |
| **Plasmids allowing mutant construction** | |  |
| TRO186 | AAATGTGCGCGGAACCCCTA | pYT313 vector |
| TRO187 | CGGCACATAACAAACAAATTGGC | pYT313 vector |
| TRO336 | TAGGGGTTCCGCGCACATTTCAGCACAGCCCAGAAATTATC | Upstream region of *hfpR* for pGi27 |
| TRO337 | GAAAATTTGGTGCAAACCAAAAGCTTTATTTATA | Upstream region of *hfpR* for pGi27 |
| TRO338 | GCTTTTGGTTTGCACCAAATTTTCGGAAAATTGCAA | Downstream region of *hfpR* for pGi27 |
| TRO339 | GCCAATTTGTTTGTTATGTGCCGTTCGTCCGAATTATAGCCTGTT | Downstream region of *hfpR* for pGi27 |
| TRO358 | TAGGGGTTCCGCGCACATTTcgctagctttaacacacat | Upstream region of *hfpY*  for pGi27 |
| TRO359 | ctcacgccacatttatattacataatgaaagtgttaaaatataaatg | Upstream region of *hfpY* for pGi27 |
| TRO360 | catttatattttaacactttcattatgtaatataaatgtggcgtgagatag | Downstream region of *hfpY* for pGi27 |
| TRO361 | GCCAATTTGTTTGTTATGTGCCGgaggcaatataggaactgctg | Downstream region of *hfpY* for pGi27 |
| TRO408 | TAGGGGTTCCGCGCACATTTGTCGAGTTTCACATCGTTATTGA | Upstream region of *bfpR* for pGi38 |
| TRO409 | CTCCCAAAACCATAGTATAGGGTAATTTTTATAGCGTTC | Upstream region of *bfpR* for pGi38 |
| TRO410 | ACCCTATACTATGGTTTTGGGAGTTAGTTTCTTCTTATAA | Downstream region of *bfpR* for pGi38 |
| TRO411 | CAATTTGTTTGTTATGTGCCGTGGCAAAGAATTTTTACGGATTA | Downstream region of *bfpR* for pGi38 |
| **Plasmids allowing chromosomal gene integration** | |  |
| TRO390 | ATGCAAGCTTGGCGTAATC | pGi37 vector |
| TRO419 | CCGCTGCATAACCCTGCTT | pGi37 vector |
| TRO393 | GGTGCGAGGATCCTCCTAACGCAAAATACTGTAATTAGTA | Upstream of insertion site for pGi37 |
| TRO420 | GAAGCAGGGTTATGCAGCGGTCAAATTACATCATCATAAGTCCA | Upstream of insertion site for pGi37 |
| TRO396 | AAGCGGGCTTATTGCTGCAGATTGAAGTGAAAATCATTTTTTTTAAG | Downstream of insertion site for pGi37 |
| TRO397 | GATTACGCCAAGCTTGCATAGAAACACACGATAAAAAAGAAATAAC | Downstream of insertion site for pGi37 |
| TRO394 | GTTAGGAGGATCCTCGCACCACACTTTTTCAAAC | P*_rpsL_*-mChFp K7 for pGi37 |
| TRO395 | CTGCAGCAATAAGCCCGCTTTTGCG | P*_rpsL_*-mChFp K7 for pGi37 |
| TRO245 | CTCCTTTGCTTACCATAATTAAATACTAAAAATTACTTGTTTATAAA | pCP23 fragment for pGi19 |
| TRO246 | caaataggcctagctgactttcc | pCP23 fragment for pGi19 |
| TRO247 | ATTTTTAGTATTTAATTATGGTAAGCAAAGGAGAAGAAGATAACATG | mChFp gene for pGi19 |
| TRO248 | AAAGTCAGCTAGGCCTATTTGTATAATTCATCCATTCCTCCG | mChFp gene for pGi19 |
| TRO125 | GTCAGCTAGGCAATTAAATACTAAAAATTACTTGTTTATAAAAT | pCP23 fragment for pCP23-P*_rpsL_* |
| TRO126 | TTAGTATTTAATTGCCTAGCTGACTTTCCGC | pCP23 fragment for pCP23-P*_rpsL_* |
| TRO108 | GTGTGGTGCGATGAACTATCGCTTTGCGTCG | P*_rpsL_* fragment for pCP23-P*_rpsL_* |
| TRO111 | AAAAGCGGGCTTATTGCTCGGTCTTGCCTTGCTC | P*_rpsL_* fragment for pCP23-P*_rpsL_* |
| TRO443 | GCCTAGCTGACTTTCCGC | plasmid fragment for pGi39 |
| TRO444 | AATTAAATACTAAAAATTACTTGTTTATAAAATTCC | plasmid fragment for pGi39 |
| TRO421 | gcggccgccgactagtgagctcgtcgacccggggcctagctgactttccgc | MCS sequence for pGi39 |
| TRO422 | cccgggtcgacgagctcactagtcggcggccgcaattaaatactaaaaattacttgtttataaaattcc | MCS sequence for pGi39 |
| TRO434 | aattaaatactaaaaattacttgtttataaaattcccgc | plasmid fragment for pGi42 |
| TRO435 | gcctagctgactttccgcc | plasmid fragment for pGi42 |
| TRO436 | ataaacaagtaatttttagtatttaattATGACAACAACCAAAAAAACCA | FP0506 fragment for pGi42 |
| TRO437 | gggcggaaagtcagctaggcTTATTGTAGATTAAAGTATTCTTGATCAGAC | FP0506 fragment for pGi42 |
| TRO404 | GCGATTTTGTTCTAATCACCCCAAA | chromosomal structure analysis |
| TRO405 | TGAGGTTTGGGTTTAAGAAAGCCAAA | chromosomal structure analysis |
| TRO402 | GAGCATGATAATTTAGTAGCAGAAGCTGGAG | chromosomal structure analysis |
| TRO403 | TCCTTAAGATGTACAGGCGCAAGAACC | chromosomal structure analysis |
|  |  |  |
| **pGi39 derivative plasmids allowing ectopic complementation of mutants** | | |
| TRO443 | GCCTAGCTGACTTTCCGC | pGi39 vector |
| TRO445 | GGATCCTCCTAACGCAAAATAC | pGi39 vector |
| TRO446 | GTATTTTGCGTTAGGAGGATCCTCGCTAAAGTTAAACTTTACTTCAT | P*_hfpR_*-*hfpR* for pGi44 |
| TRO447 | GGCGGAAAGTCAGCTAGGCTTAAAAATTAAATTGCAATTTTCCGAA | P*_hfpR_*-*hfpR* for pGi44 and pGi46 |
| TRO448 | GTATTTTGCGTTAGGAGGATCCTCCTCCTAGTTTTATGTTTTAATTTATTT | P*_bfpR_1_*_-_*bfpR* for pGi45 and pGi46 |
| TRO449 | GGCGGAAAGTCAGCTAGGCTTATAAGAAGAAACTAACTCCCAAAAC | P*_bfpR_1_*_-_*bfpR* for pGi45 |
| TRO452 | gtttaactttagcgaTTATAAGAAGAAACTAACTCCCAAAAC | P*_hfpR_*-*hfpR* for pGi46 |
| TRO453 | GTTAGTTTCTTCTTATAAtcgctaaagttaaactttacttcat | P*_bfpR_1_*-*bfpR* for pGi46 |
| TRO519 | cattcttaattattttctgcaaatatatgtcc | P*_hfpR_* for pGi55 |
| TRO520 | tcattttaagcctagctgactttccg | P*_hfpR_* for pGi55 |
| TRO521 | ggacatatatttgcagaaaataattaagaatgaaaaatcatttttttaaagttgcag | *hfpY* for pGi55 |
| TRO522 | gaaagtcagctaggcttaaaatgatgtcgtgctattagc | *hfpY* for pGi55 |
|  |  |  |
| **Construction of pCP*Gm*^r^ derivative replicative plasmids** | | |
| TRO454 | ACATCGACACCAGAACTATCGCTTTGCGTCGTTC | pCP*Gm*^r^ vector |
| TRO455 | ATTATACAAATAGGAAAAGGCTCCTGTTTTTGAGC | pCP*Gm*^r^ vector |
| TRO456 | GCGATAGTTCTGGTGTCGATGTAAGAAATCAAGTT | *mCherry* gene for pGi47 |
| TRO457 | CTCGAGACTAGTCTTAAGCGGCCGCTAGCCACTGTTTGCTAAGTGAGCTAGAC | *mCherry* gene for pGi47 |
| TRO458 | CCGCTTAAGACTAGTCTCGAGAAAAATTATGGTAAGCAAAGGAGAAGAAGATAA | Rho independent terminator for pGi47 |
| TRO459 | AACAGGAGCCTTTTCCTATTTGTATAATTCATCCATTCCTCCG | Rho independent terminator for pGi47 |
| TRO460 | CTCGAGAAAAATTATGGTAAGCAAAGG | pGi47 vector |
| TRO461 | ACTAGTCTTAAGCGGCCGCTA | pGi47 vector |
| TRO462 | TAGCGGCCGCTTAAGACTAGTTCGCTAAAGTTAAACTTTACTTCAT | P*_hfpR_* for pGi48 |
| TRO463 | CCTTTGCTTACCATAATTTTTCTCGAGTCTTAATTATTTTCTGCAAATATATGTCC | P*_hfpR_* for pGi48 |
| TRO464 | TAGCGGCCGCTTAAGACTAGTTCCTCCTAGTTTTATGTTTTAATTTATTT | P*_bfpR_1_* for pGi49 |
| TRO465 | CCTTTGCTTACCATAATTTTTCTCGAGAGTATAGGGTAATTTTTATAGCGTTC | P*_bfpR_1_* for pGi49 |
| TRO789 | TAGCGGCCGCTTAAGACTAGTCTCATTATTAGCGATGGTAGTATTG | P*_bfpR_2_* for pGi74 |
| TRO790 | CCTTTGCTTACCATAATTTTTCTCGAGAGTATAGGGTAATTTTTATAGCGTTC | P*_bfpR_2_* for pGi74 and P*_bfpR_3_* for pGi80 |
| TRO824 | TAGCGGCCGCTTAAGACTAGTGGTGTTGTGGTACAAGATAATAATGATAC | P*_bfpR_3_* for pGi80 |
|  |  |  |
| **RT-qPCR** | | |
| TRO497 | GGTCTACGCACTTGCGTTCT | Housekeeping gene *rpsA* forward |
| TRO498 | GTCCAAGGGTCTTGCGTCAT | Housekeeping gene *rpsA* reverse |
| TRO499 | TAGTGTTCCGCCACTTACCG | Housekeeping gene *frr* forward |
| TRO500 | CCTATTTTCGCGTCTTCGGC | Housekeeping gene *frr* reverse |
| TRO591 | GCTGGCTTTAGGTACGATAATCA | *hfpR* forward |
| TRO592 | TCTGCCACGTTATACCCCAA | *hfpR* reverse |
| TRO593 | ACGTTGAGGTTTTGAAGATGGA | *THC0290*_*1813* forward |
| TRO594 | AAACTTCGACCAACTCTGCG | *THC0290*_*1813* reverse |
| TRO595 | GTAAACGGAGGTGCTGCAAT | *hfpY* forward |
| TRO596 | CCAACCATTACCGCTCCCTA | *hfpY* reverse |
| TRO597 | TCATGCAACTTCAAGATTGGTTT | *THC0290*_*1811* forward |
| TRO598 | GAATTGGCTTCGATTTTAAATTTCAAAA | *THC0290*_*1811* reverse |
| TRO599 | CGGGAATCGTGTTTTGGTGT | *bfpR* forward |
| TRO600 | CCAACGCATCAGAGCCATAC | *bfpR* reverse |
| TRO863 | GCGATGGTAGTATTGTTTTCGAT | *THC0290*_*1678* forward |
| TRO864 | GTGGGTAATTTGAGAACCAGAA | *THC0290*_*1678* forward |
|  |  |  |
| **3’-5’ RACE experiments** | | |
| TRO806 | CCCGCACTTCGCCCCACAA | Reverse transcription *hfpR* |
| TRO807 | GGGAGCGGTAATGGTTGGTACAA | PCR1 *hfpR* and *hfpY* forward |
| TRO808 | CCCCACAAGTGGCACACCAT | PCR1 *hfpR* reverse |
| TRO809 | CTCCTCTTGCCGGAAAAGTATTTGT | PCR2 *hfpR* and *hfpY* forward |
| TRO810 | CCTTCTGCGCCACCAAAATCT | PCR2 *hfpR* reverse |
| TRO811 | CCCGATACAGGTCCAAACGACATA | Reverse transcription *hfpY* |
| TRO812 | CGACATACCTACTTGTGGTGCATT | PCR1 *hfpY* reverse |
| TRO813 | GTGGTTGCGGAGTAAGAACAG | PCR2 *hfpY* reverse |
| TRO814 | GCCAATCCCTCCATGAGCGTT | Reverse transcription *bfpR* |
| TRO815 | CGAAACCGTAACTGGTCTGAAAGA | PCR1 *bfpR* forward |
| TRO816 | GCGGCTCCTTTTTGTTGTAATTCT | PCR1 *bfpR* reverse |
| TRO817 | CGCTGGTAAATGTAGGTTTAGGTGGAA | PCR2 *bfpR* forward |
| TRO818 | CTCAGGAATAGAAACCAAGACAGTTTCT | PCR2 *bfpR* reverse |
| TRO825 | CACCCGAACAAACAAAACCACCT | PCR1 *THC0290_1678* reverse |
| TRO826 | GGTGTTGGTGGGTAATTTGAGAACCA | PCR2 *THC0290_1678* reverse |
| TRO827 | GCCCATTCCAGAGAAGTTTCTCCA | PCR1 *asd* reverse |
| TRO828 | CTCCACCAGCAGAAAACAAAGCAA | PCR2 *asd* reverse |
|  |  |  |
| **OVERLAPPING RT-PCR** | | |
| TRO672 | GGGCATAGCGAAGCGG | *asd* forward |
| TRO673 | GCTCATAACTATGCACCGATTG | *THC0290_1678* reverse |
| TRO674 | GTTCGGGTGTTTCTTGTTTTC | *THC0290_1678* forward |
| TRO675 | CCACTTCAATCCCTCACGC | *bfpR reverse* |
| TRO676 | GAAAAGCCCGACCCGTAG | *upstream of bfpR forward* |
| TRO677 | ATTCCCGCTTAACCCACG | *bfpR* reverse |

T3. Genomic organization and predicted protein functions of the iron-induced gene cluster encoding the Hfp system in strain OSU THCO2-90.

^(a)^ Gene orientation on the chromosome (↑, forward strand; ↓ reverse strand) and ^(b)^differential expression data were retrieved from Guérin *et al* [2] ([https://fpeb.migale.inrae.fr](https://fpeb.migale.inrae.fr/)) that provides microarray expression level along the genome, transcription start sites (TSSs) and differential expression analyses for diverse biological conditions. ^(a)^ Arrows indicate the transcriptional orientation of each gene cluster (TU); ^(b)^ Genes significantly upregulated (yellow) and down-regulated (blue) in iron-limited condition (‘**DIP**’**;** TYES 2,2’-dipyridyl 25 µM), under rainbow trout plasma exposure (‘**Plasma**’) or in the presence of blood (‘**Blood**’; TYES agar supplemented with 10% horse blood) are indicated. Values are log2-fold changes of the test condition compared to the relevant control condition.

Supplementary references

1. Zhu Y, Thomas F, Larocque R, et al. Genetic analyses unravel the crucial role of a horizontally acquired alginate lyase for brown algal biomass degradation by *Zobellia galactanivorans*. Environ Microbiol. 2017 Jun;19(6):2164-2181.

2. Guérin C, Lee B-H, Fradet B, et al. Transcriptome architecture and regulation at environmental transitions in flavobacteria: the case of an important fish pathogen. ISME Communications. 2021 2021/07/07;1(1):33.

3. Rochat T, Pérez-Pascual D, Nilsen H, et al. Identification of a novel elastin-degrading enzyme from the fish pathogen *Flavobacterium psychrophilum*. Applied and environmental microbiology. 2019 Jan 11.

4. Pei J, Kim BH, Grishin NV. PROMALS3D: a tool for multiple protein sequence and structure alignments. Nucleic acids research. 2008 Apr;36(7):2295-300.

5. Ferrieres L, Hemery G, Nham T, et al. Silent mischief: bacteriophage Mu insertions contaminate products of *Escherichia coli* random mutagenesis performed using suicidal transposon delivery plasmids mobilized by broad-host-range RP4 conjugative machinery. Journal of bacteriology. 2010 Dec;192(24):6418-27.

6. Bertolini JM, Wakabayashi H, Watral VG, et al. Electrophoretic detection of proteases from selected strains of *Flexibacter psychrophilus* and assesment of their variability. Journal of aquatic animal health. 1994;6:224-233.

7. Pérez-Pascual D, Rochat T, Kerouault B, et al. More than gliding: Involvement of GldD and GldG in the virulence of *Flavobacterium psychrophilum*. Frontiers in microbiology. 2017;8:2168.
